# Physical and Mechanical Characterisation of 3D-Bioprinted Hydrogels for Dental Applications: A Scoping Review

**DOI:** 10.3390/gels12060524

**Published:** 2026-06-11

**Authors:** Nur Haziqah Junaidi, Nurulhuda Mohd, Noor Hayaty Abu Kasim, Masfueh Razali

**Affiliations:** 1 Department of Restorative Dentistry, Faculty of Dentistry, Universiti Kebangsaan Malaysia, Jalan Raja Muda Abdul Aziz, Kuala Lumpur 50300, Malaysia; 2 Oral Health Division, Putrajaya 62590, Malaysia; 3 Department of Restorative Dentistry, Faculty of Dentistry, University of Malaya, Kuala Lumpur 50603, Malaysia; 4 Mesomorph Worldwide Sdn. Bhd., Ltd., Kuala Lumpur 52200, Malaysia

**Keywords:** 3D bioprinting, hydrogel, dental tissue regeneration, mechanical properties, physical characterisation

## Abstract

Recent advances in three-dimensional (3D)-bioprinted hydrogels show promise for overcoming the limitations of conventional techniques for dental tissue regeneration. This scoping review systematically analyses the physical, mechanical, and rheological properties of these hydrogels in dental applications, aiming to identify knowledge gaps, limitations, and current and future directions for advancing and translating hydrogel-based 3D bioprinting in dentistry. In accordance with PRISMA-ScR guidelines, a comprehensive literature search was conducted across Ovid, PubMed, EBSCOhost, and Web of Science up to January 2026. Included studies focused on (i) 3D-bioprinted hydrogels, (ii) quantitative characterisation, and (iii) dental tissue engineering. A total of twenty-one studies met the inclusion criteria. The findings revealed substantial variability in formulations and properties. Gelatine-based hydrogels reinforced with β-tricalcium phosphate demonstrated the highest compressive strength within the range of cancellous bone, whereas GelMA/PEGDA composites exhibited tunable stiffness suitable for soft tissue applications. Extrusion-based bioprinting emerged as the predominant method, with photocrosslinking and ionic crosslinking as the primary gelation techniques. Biodegradation rates varied notably with composition and regenerative objectives. This review uniquely consolidates the physical, mechanical, and rheological evaluations of 3D-bioprinted hydrogels for dental applications. The review highlights critical gaps in methodological standardisation and validation, emphasising the importance of biomaterial selection to optimise scaffolds and regenerative outcomes in periodontal, bone, and pulp tissue engineering.

## 1. Introduction

Globally, the shortage of tissues and organs remains a significant challenge in healthcare [1]. The field of dentistry shares an overarching goal with medicine: restoring the optimal form and function of natural tissues through healing and regeneration. For instance, in patients with periodontitis, current treatment approaches such as guided tissue regeneration and bone grafting are typically limited to selected cases depending on the morphology and size of osseous defects [2]. However, these conventional therapies present several limitations, including difficulty in achieving precise adaptation and conformation to complex defect geometries [3,4].

**Figure 1:**
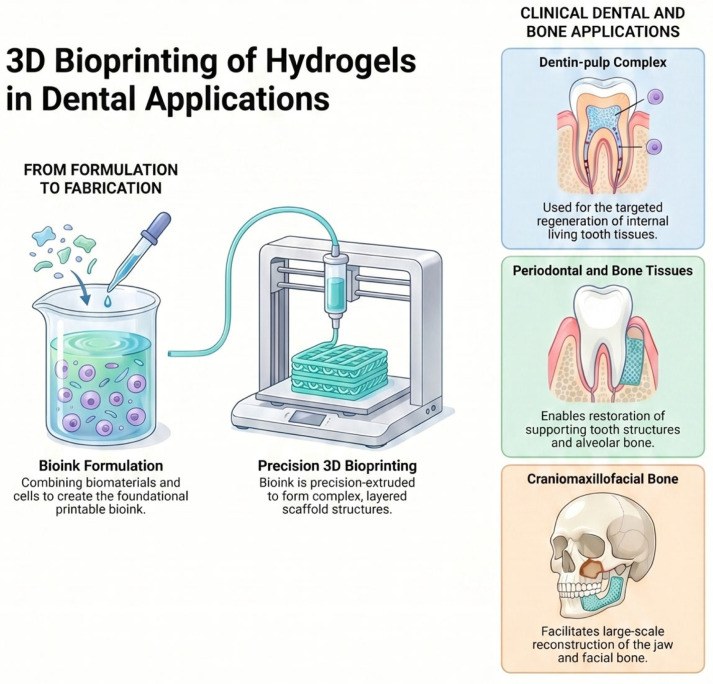
3D bioprinting strategy for dental applications, including regeneration of the dentine–pulp complex, periodontal tissues, alveolar bone tissues, and craniomaxillofacial bone. Image generated using NotebookLM.

To address these challenges, advanced manufacturing technologies have been developed to fabricate scaffolds that closely mimic the architecture of native biological tissues. Among these, bioprinting has emerged as a promising approach, enabling the three-dimensional (3D) printing and spatial patterning of cells and biomaterials to generate dental tissue-like structures, as shown in Figure 1. This technology has opened new avenues in tissue engineering, regenerative medicine, and dentistry.

3D bioprinting is considered a transformative technology capable of constructing living tissues with precise anatomical control and defined cellular composition. The concept originated from stereolithography, which was first described by Charles W. Hull in 1984 [5]. Since then, bioprinting has evolved into a versatile platform for fabricating customised constructs with complex architectures, thereby addressing key challenges in regenerative applications.

**Figure 2:**
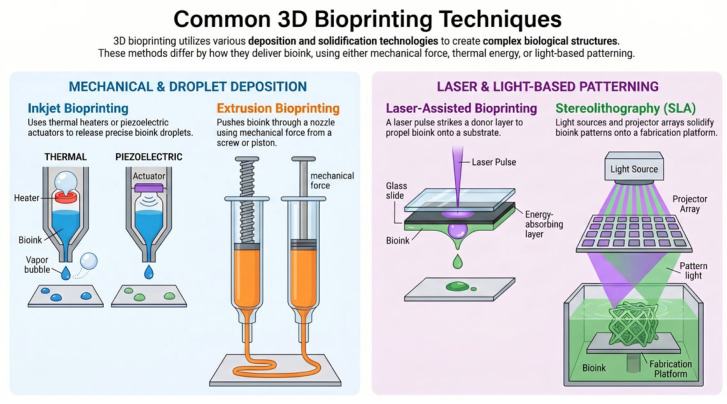
Common 3D bioprinting techniques, which include inkjet bioprinting, extrusion bioprinting, laser-assisted bioprinting, and stereolithography. Image generated using NotebookLM.

Several 3D bioprinting techniques are currently employed, including extrusion-based [6,7], inkjet-based [8], laser-assisted [9], and stereolithography (SLA) [10] approaches, as shown in Figure 2. Extrusion-based bioprinting, commonly used in dental applications, uses pneumatic, piston, or screw-driven systems to deposit bioinks, making it particularly effective for viscous materials [11]. It is preferred because it can use a wide variety of biomaterials to create multilayered scaffolds in tissue engineering [12]. Additionally, it can handle high cell densities, a range of material viscosities, and different crosslinking mechanisms [13]. In contrast, inkjet-based bioprinting uses a drop-on-demand process with thermal or piezoelectric printheads, offering advantages such as low cost and high speed, though it can clog at high cell densities [14]. Laser-assisted bioprinting uses a laser as the energy source and involves an energy-absorbing layer, a donor ribbon, and a receiving substrate [15]. This non-contact, nozzle-free method enables the deposition of high-viscosity bioinks with high resolution and cell viability, without clogging [14]. However, the effects of lasers on cells remain uncertain [16]. Meanwhile, SLA employs ultraviolet light or electron beams to trigger polymerisation, allowing for the creation of complex structures with very high resolution. However, it is limited by slow printing speeds, high costs, and a narrow selection of materials with appropriate processing characteristics [17].

Although significant advances have been made, the clinical application of 3D bioprinting is still limited by challenges such as producing large, vascularised constructs that can be implanted. For successful tissue regeneration, scaffolds must not only replicate the defect’s anatomical structure but also support cell delivery, neovascularisation, and tissue remodelling. Ideal scaffolds should have a highly porous, interconnected 3D architecture that facilitates cell attachment, migration, proliferation, and nutrient exchange, while also being biocompatible and biodegradable at a controlled rate to match tissue growth [18].

Advances in biomaterials science, cell biology, and immunology have substantially contributed to the development of more sophisticated scaffolds. An enhanced understanding of host immune responses [19,20] and mechanobiological interactions between cells and their extracellular matrix [21,22] has led to the design of biomaterials that better integrate with host tissues. Nevertheless, replicating the full complexity of native tissues and providing an optimal microenvironment for regeneration remain considerable challenges [23,24].

Hydrogels have emerged as a promising class of biomaterials for tissue engineering and have also gained increasing attention in dental applications. First introduced by Wichterle and Lím in 1960, hydrogels are 3D polymeric networks formed by physical or chemical crosslinking, capable of retaining large amounts of water while exhibiting soft, elastic properties [25,26,27]. Due to their structural similarities to extracellular matrices (ECMs), versatility, and high tunability, hydrogels are well suited to mimicking native tissue environments. They are generally biocompatible and biodegradable, enabling gradual scaffold degradation while supporting new tissue formation [28,29]. Furthermore, they have adjustable physical, mechanical, and rheological properties, allowing customisation of stiffness, porosity, degradation rate, and printability for particular dental uses [30]. These properties make hydrogels highly suitable for regenerating periodontal tissues, alveolar bone, dental pulp, and oral soft tissues [31,32].

In 3D bioprinting, hydrogels exhibit shear-thinning and viscoelastic properties that facilitate extrusion during printing and support structural stability afterwards [33]. Additionally, hydrogels serve as carriers for bioactive molecules, stem cells, antimicrobial agents, growth factors, and nanoparticles, thereby enhancing osteogenic, angiogenic, and immunomodulatory responses [26]. Adding bioactive fillers like hydroxyapatite, bioactive glass, graphene oxide, and nanocellulose can enhance the mechanical strength and biological performance of hydrogel scaffolds [34,35,36].

Hydrogels can be broadly categorised into natural, semi-synthetic, or synthetic polymers [37]. Each has distinct advantages and drawbacks for dental tissue engineering applications. Natural polymers such as gelatine and collagen offer superior biocompatibility and inherent cell-adhesive properties, whereas synthetic polymers provide greater control over mechanical properties [38]. Semi-synthetic hydrogels combine the advantages of both, enabling the development of advanced biomaterials tailored to specific applications [39].

Natural hydrogels such as gelatine, collagen, alginate, hyaluronic acid, chitosan, and gelatine methacryloyl (GelMA) are widely favoured for their excellent biocompatibility, biodegradability, and resemblance to the native ECM [40,41]. These properties promote favourable cell adhesion, proliferation, migration, and differentiation, making them highly suitable for the regeneration of periodontal, pulp, and bone tissues [42,43]. However, natural hydrogels often exhibit poor mechanical strength, rapid degradation, limited structural stability, and reduced print fidelity, which may compromise their ability to withstand the biomechanical forces in the oral environment [44].

In contrast, synthetic hydrogels, such as polylactic acid (PLA), polycaprolactone (PCL), polyethylene glycol diacrylate (PEG/PEGDA), and polylactic-co-glycolic acid (PLGA), offer superior mechanical strength, tunable degradation profiles, improved printability, and greater reproducibility during fabrication [45]. Their physicochemical properties can be finely tuned to achieve desirable rheological behaviour, scaffold architecture, and degradation rates [46]. Nevertheless, synthetic hydrogels generally lack the inherent biological cues required for optimal cellular attachment and tissue integration, potentially reducing their bioactivity and regenerative potential when used alone. In addition, some synthetic materials may exhibit reduced biocompatibility or require further surface modifications to enhance cellular interactions [47,48].

To overcome the limitations of individual hydrogel classes, hybrid hydrogels that combine natural and synthetic biomaterials have emerged as a promising strategy for dental bioprinting applications [45]. Hybrid systems aim to integrate the favourable biological properties of natural hydrogels with the superior mechanical stability and printability of synthetic polymers. For example, combining GelMA with PEG or incorporating nanomaterials such as hydroxyapatite, bioactive glass, graphene oxide, or nanocellulose may enhance scaffold stiffness, osteoconductivity, print fidelity, and structural integrity while preserving cytocompatibility [49]. Consequently, hybrid hydrogels demonstrate greater versatility and may more closely mimic the hierarchical complexity of dental tissues [50]. These systems also enable more predictable modulation of degradation behaviour, mechanical performance, and the delivery of bioactive molecules [51,52].

Despite these advantages, challenges remain in optimising the balance among bioactivity, mechanical stability, degradation rates, and printability in hybrid constructs [53]. Excessive reinforcement may diminish cell viability or hinder hydrogel extrusion behaviour, whereas inadequate reinforcement could compromise structural integrity [54]. Furthermore, the clinical translation of 3D-bioprinted hydrogel-based scaffolds in dentistry presents several significant biosafety challenges. A primary concern is biocompatibility, as certain biomaterials, crosslinking agents, nanoparticles, or degradation by-products may provoke cytotoxicity, inflammatory responses, or foreign-body reactions following implantation. Additionally, uncontrolled biodegradation may compromise scaffold stability or hinder tissue remodelling and cellular infiltration. The oral environment further complicates scaffold performance due to exposure to saliva, fluctuating pH, microbial contamination, and mechanical loads, all of which may adversely affect long-term regenerative outcomes. Hydrogel scaffolds with high water content may favour bacterial colonisation if antimicrobial properties are inadequate [55].

Another significant challenge concerns limitations in vascularisation and the incorporation of bioactive components such as stem cells and growth factors [26]. Insufficient vascularisation within larger constructs may impair oxygen and nutrient diffusion, leading to poor cell survival and tissue integration. Furthermore, cell-laden bioprinted scaffolds may pose risks of abnormal cellular differentiation, genetic instability, or unpredictable host responses [26].

Several mitigation strategies have been proposed to address these biosafety concerns. These include incorporating antimicrobial agents, angiogenic factors, immunomodulatory molecules, and controlled drug delivery systems, which may further enhance scaffold safety and regenerative outcomes [52]. More long-term in vivo validation studies and standardised biosafety assessment protocols should be conducted, as these remain limited [2].

**Figure 3:**
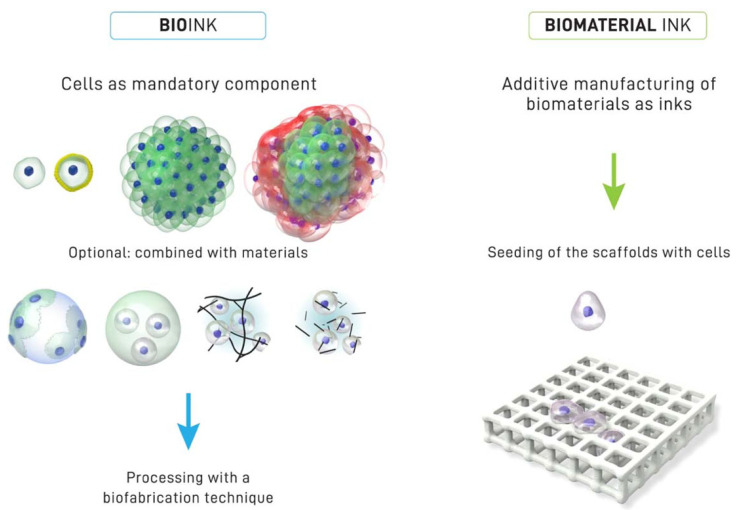
The characteristic distinction between bioink and biomaterial ink. Reproduced with permission [56]. Copyright 2018 IOP publishing under a Creative Commons Attribution 3.0 Unported (CC BY 3.0), https://creativecommons.org/licenses/by/3.0/ (accessed on 25 May 2026).

In tissue engineering, cells can be incorporated into hydrogel-based scaffolds either by post-fabrication seeding or by embedding them directly in the bioink (cell-laden systems), as shown in Figure 3. Cell seeding involves introducing cells onto a pre-prepared scaffold, whereas cell-laden approaches encapsulate cells within the hydrogel matrix during fabrication, resulting in a more uniform cell distribution. Cell seeding can be performed using techniques such as pipetting, whereas cell-laden constructs are usually produced by mixing living cells with a biocompatible matrix or scaffold material [56]. Cell printing technology has emerged as a preferred biofabrication approach compared with conventional methods of seeding cells onto pre-formed scaffolds. 3D bioprinting enables the direct incorporation of living cells into printed constructs, allowing improved spatial control and distribution of cells. In contrast, traditional cell seeding approaches may result in significant cell loss during seeding, which can compromise cell viability and overall biological performance [57].

**Figure 4:**
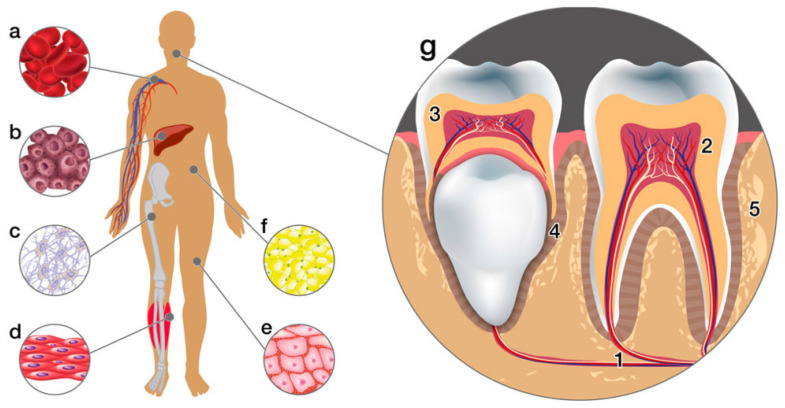
Sources of mesenchymal stem cells (MSCs), also called “universal cells”. The image shows human tissue sources: (**a**) peripheral blood, (**b**) liver, (**c**) bone marrow, (**d**) muscles, (**e**) skin, (**f**) adipose tissue, and (**g**) dental tissues. (1. apical dental papilla, 2. dental pulp; 3. pulp from the exfoliated deciduous tooth, 4. periodontal ligament, and 5. alveolar bone) [59].

Mesenchymal stem cells (MSCs), often called “universal cells,” are highly valued for tissue regeneration because of their ability to self-renew and differentiate into various functional cell types in response to appropriate signals [58]. They can be obtained from both embryonic and adult sources [59] and are readily isolated from tissues such as bone marrow, adipose tissue, umbilical cord, placenta, and dental tissues, as shown in Figure 4. In the oral cavity, dental stem cells are found in the periodontal ligament (PDLSCs), dental pulp (DPSCs), apical papilla (SCAPs), and exfoliated deciduous teeth (SHEDs) [58], showing their great potential for oral tissue engineering [60] (see Figure 4). Additionally, MSCs have immunomodulatory properties and release bioactive factors that aid in tissue repair and regeneration [61]. In addition to MSCs, various other cell types are also used in 3D bioprinting for dental and craniofacial applications. These include fibroblasts for extracellular matrix and soft tissue development, osteoblasts for bone regeneration, and endothelial cells for vascularisation. Chondrocytes are used in cartilage-related applications, whereas induced pluripotent stem cells (iPSCs) provide an alternative source with high differentiation potential [62]. Combining different cell types in bioprinted scaffolds increases structural and functional complexity, thereby more closely mimicking the native tissue environment.

Each bioprinting procedure requires a specific set of rheological, physical, and mechanical properties of the bioink to produce a successful bioprinted structure. For instance, extrusion-based processes must control shear-thinning properties, such as viscosity and shear stress, to minimise cell damage. This review reports and analyses natural and synthetic materials used to produce hydrogels with different features and behaviours for dental applications. There remains limited literature on the use of 3D-bioprinted hydrogels for dental applications. Thus, this scoping review aimed to identify existing knowledge gaps and address the following questions: (i) How have 3D-bioprinted hydrogels been developed for use in dentistry? (ii) What types of biomaterials are used to produce 3D-bioprinted hydrogels? (iii) What are the physical, mechanical, and rheological properties of 3D-bioprinted hydrogels developed for dental applications?

Understanding these properties is essential, as dental tissues such as bone and the periodontal ligament possess distinct biomechanical characteristics that must be replicated to achieve successful tissue regeneration. This scoping review aims to map and synthesise the current evidence on 3D-bioprinted hydrogels developed for dental applications, with particular emphasis on the biomaterials used, their fabrication methods, and their physical, mechanical, and rheological properties. Additionally, this review seeks to identify existing knowledge gaps, limitations in current research, and potential future directions for the advancement and clinical translation of hydrogel-based 3D bioprinting in dentistry.

To the best of our knowledge, this review is the first comprehensive, biomaterial-focused synthesis that integrates the physical, mechanical, and rheological characterisation of 3D-bioprinted hydrogels across multiple dental applications. The novelty of this review lies in its comparative assessment of how biomaterial selection and reinforcement strategies influence scaffold performance and printability, highlighting the potential for predictable modulation of hydrogel properties. Furthermore, this review identifies critical translational gaps between in vitro material characterisation and clinical tissue regeneration outcomes. Key priorities, including standardisation of testing protocols, long-term in vivo validation, and integration of computational modelling approaches, are also discussed to facilitate the future clinical translation of 3D-bioprinted hydrogel scaffolds. Ultimately, this work aims to provide valuable insights to guide the development of optimised hydrogel-based scaffolds and advance their application in regenerative dentistry.

## 2. Materials and Methods

### 2.1. Search Strategy

This review applied the methodological framework from the Joanna Briggs Institute guidelines for scoping reviews and was conducted in accordance with the Preferred Reporting Items for Systematic Reviews and Meta-Analyses extension for Scoping Reviews (PRISMA-ScR) [63]. The research questions for this review are as follows: (i) How have 3D-bioprinted hydrogels been developed for use in dentistry? (ii) What types of biomaterials are used to produce 3D-printed hydrogels? (iii) What are the physical and mechanical properties of 3D-printed hydrogels developed for dental applications?

A search of the literature published through January 2026 was conducted using four databases: Ovid, PubMed, EBSCOhost, and Web of Science. The following terms were used: (“3D bioprinting” OR “3D-bioprint*” OR “3D print*” OR “3D-print*” OR “Bioprinting” OR “Three-dimensional bioprint*”) AND (“hydrogel*”) AND (“physical” OR “mechanical”) AND (“dental” OR “dentistry”). Additional records were identified through a manual search of the reference lists. The search was limited to English and had no restriction on the publication year.

### 2.2. Study Selection

The initial screening of the identified studies was conducted by two independent reviewers (N.H.J and N.M) based on the information in the titles and abstracts. In addition, the full texts of potentially eligible studies were retrieved for further screening of their suitability, as determined by the inclusion and exclusion criteria. Any disagreement between reviewers on study selection was resolved through discussion between the two.

The inclusion criteria for the included studies were defined based on the Participant/Population (P): 3D-bioprinted hydrogel; Concept (C): physical and mechanical assessments; and Context (C): dental application.

### 2.3. Data Extraction and Analysis

Extraction and synthesis of information from the included studies were summarised and presented in a table of evidence by the first reviewer (N.H.J.) and verified by the second reviewer (N.M.) to ensure alignment with the research questions. The extracted data from the included studies comprised publication details (first author, year of publication, and country of study), 3D bioprinting strategy (type of 3D bioprinter and parameters of the 3D printing technique), type of hydrogels, crosslinking type, gelation time and temperature, dimensions of the hydrogel, application in the dental field, and outcomes of the 3D bioprinting (physical and mechanical assessments).

## 3. Results

### 3.1. Study Selection and Characteristics

**Figure 5:**
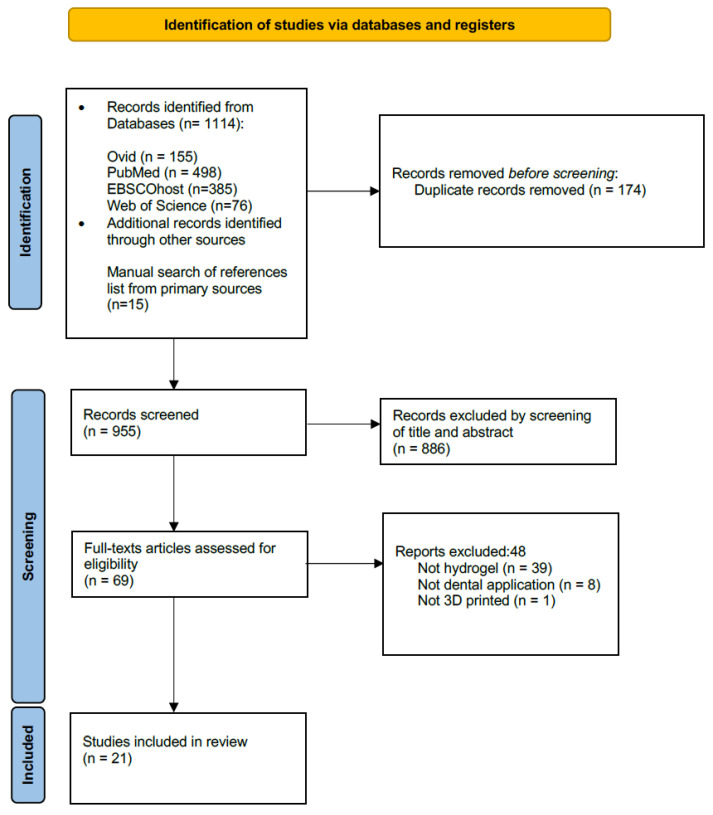
PRISMA flow diagram depicting the results of the search strategy.

This revised search strategy generated 1114 records from four databases through January 2026: Ovid (*n* = 155), PubMed (*n* = 498), EBSCOhost (*n* = 385), and Web of Science (*n* = 76). In addition to electronic databases, a manual search of reference lists from primary sources was carried out, and additional eligible studies were added (*n* = 15). Of these, 174 duplicates were excluded, and 955 records were assessed based on their titles and abstracts. This was performed using the online literature review application, Rayyan software (http://rayyan.qcri.org (accessed on 31 January 2026)). Moreover, full texts of 69 articles were retrieved for eligibility assessment against the inclusion and exclusion criteria. Of these, 48 were further excluded because the materials used in the studies were not hydrogel (*n* = 39), were not 3D-printed (*n* = 1), or were not for dental applications (*n* = 8). Finally, 21 articles were included in this review, as recorded in Figure 5.

### 3.2. Characteristics of Included Studies

A third of the included articles were conducted in China (*n* = 7) [64,65,66,67,68,69,70]. This was followed by Korea (*n* = 4) [71,72,73,74], the US (*n* = 3) [75,76,77], Australia (*n* = 2) [78,79], Germany (*n* = 2) [80,81], Taiwan (*n* = 1) [82], Canada (*n* = 1) [83], and Spain (*n* = 1) [84]. The number of publications rose steadily from 2017 to 2026, reflecting growing interest in 3D-bioprinted hydrogels for dental applications. The key characteristics of the included studies are summarised in Table 1.

Nearly all the research discussed in this review employed extrusion-based 3D bioprinting to create hydrogel-based scaffolds. The extrusion-based method was used in five studies for bone regeneration [64,66,71,72,75], three studies focused exclusively on periodontal tissues [69,78,79], two studies used it for bone and dentine [73,76], and two studies used it for general dental tissue regeneration [68,80]. Following which, one study used extrusion-based 3D bioprinting for a combination of bone, dentine, and pulp [77]; one study for periodontal ligament, dentine, and pulp [74]; one study for bone and cementum [65]; one study for bone and periodontal [70]; one study for soft tissue in the dentoalveolar region [81]; one study for enamel only [83]; one study for dentine only [82]; and one study for pulp only [84]. One study employed digital light processing technology for alveolar bone regeneration [67]. Overall, roughly a third of these studies used hydrogel-based scaffolds for the regeneration of the cranial and alveolar bones. Figure 1 shows 3D-bioprinted hydrogels used in dental applications. The other information, such as the crosslinking types and the corresponding assessments carried out, is presented in Table 1.

**Figure 6:**
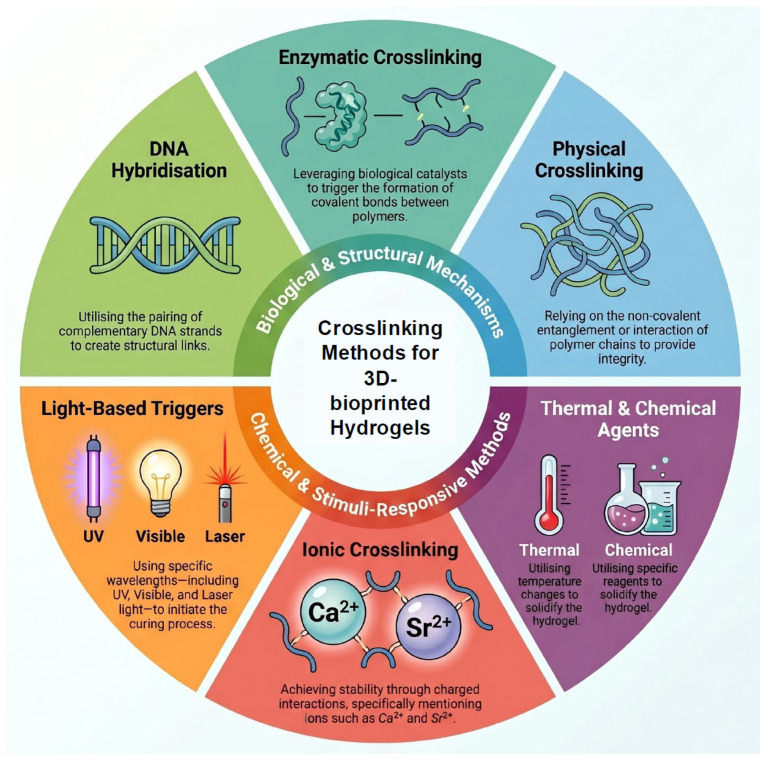
Different types of crosslinking methods for 3D-bioprinted hydrogels. Image generated using NotebookLM.

More than a third of the studies utilised photocrosslinking to crosslink their hydrogels [66,67,70,72,76,79,80,82]. This was particularly evident in GelMA-based hydrogels because of their methacrylate functional groups, which readily undergo photoinitiated polymerisation in the presence of photoinitiators, as shown in Figure 6. This enables rapid gelation, tunable mechanical properties, and precise spatial control, making GelMA highly suitable for 3D bioprinting. Consequently, photocrosslinking enhances structural integrity and printing fidelity, supporting its widespread use in scaffold fabrication for dental tissue engineering [85,86].

Another third of the studies were chemically crosslinked [64,68,69,71,73,74,78,81]. Chemical crosslinking offers a simple and effective way to improve the durability, degradation profile, and overall suitability of gelatine-based hydrogels for dental applications. It offers several advantages over other crosslinking methods, including the formation of more stable and permanent covalent bonds that enhance mechanical strength and long-term structural integrity [87]. It also enables better control over network density and degradation rates by adjusting the crosslinker concentration. Unlike photocrosslinking, it does not require light exposure or specialised equipment, making it suitable for bulk fabrication and for thicker constructs where light penetration may be limited. These features make chemically crosslinked hydrogels particularly advantageous for applications requiring sustained mechanical stability and durability [87].

Most of the studies that utilised chemical crosslinking were gelatine-based due to gelatine’s inherent chemical structure [64,71,78]. Gelatine contains abundant amino and carboxyl functional groups, which readily participate in covalent bonding with chemical crosslinkers such as genipin or glutaraldehyde [88]. This facilitates efficient network formation and enhances mechanical strength and stability. In addition, native gelatine lacks sufficient mechanical integrity and is highly water-soluble, necessitating crosslinking to maintain its structure under physiological conditions [89]. Genipin is a commonly used natural chemical crosslinker, owing to its low cytotoxicity and good biocompatibility compared with conventional agents such as glutaraldehyde [90]. Genipin forms stable covalent crosslinks with amino groups in natural polymers, thereby enhancing mechanical strength without compromising cell viability [91]. In addition, it offers controlled crosslinking rates and a reduced inflammatory response, making it particularly suitable for biomedical and dental tissue engineering applications.

### 3.3. 3D-Bioprinted Hydrogel Printing Parameters

3D bioprinting has emerged as a transformative technology for fabricating biomimetic scaffolds and tissue constructs. Researchers have employed diverse bioprinting platforms to deposit hydrogels with controlled precision, aiming to balance structural integrity, cell viability, and printability. The field integrates multiple printing methodologies, material chemistries, and process parameters to optimise scaffold fabrication outcomes, as shown in Table 2.

#### 3.3.1. Bioprinting Techniques

Extrusion-based bioprinting has become the dominant technique across nearly all studies investigated, reflecting its established reliability and ease of implementation. Of the 21 studies analysed, 20 used extrusion bioprinting [64,65,66,68,69,70,71,72,73,74,75,76,77,78,79,80], while only one study explored digital light projection (DLP) [67]. The widespread adoption of extrusion methods is reflected in the diversity of commercial and custom-built systems in use, including the Bio-Architect platform from Regenovo [78], the BioScaffolder 3.1 system from GeSiM [79], the BioX system from Cellink [72], the 3D Bioplotter from EnvisionTEC [70], and custom-engineered systems [66].

This consensus suggests that extrusion bioprinting offers practical advantages in bioink compatibility, resolution control, and reproducibility. However, the variation in nozzle geometries (ranging from 150 μm to 580 μm) and printer configurations indicates that researchers continue to optimise extrusion parameters for specific hydrogel formulations and application requirements.

#### 3.3.2. Nozzle Diameter

Nozzle size emerged as a frequently reported and carefully tuned printing parameter, with reported sizes ranging from 150 μm to 580 μm across the reviewed studies. Ma et al. (2017) employed a relatively small nozzle diameter of 150 μm at a printing pressure of 50 kPa [66], whereas Jiang et al. (2018) used a slightly larger 210 μm nozzle at an elevated pressure (0.28 MPa) [68]. Mid-range nozzle diameters of 250–300 μm were commonly reported, with Han et al. (2021) using a 300 μm nozzle [74] and Kim et al. (2022) selecting a 25G needle (approximately 250 μm inner diameter) [73]. Notably, Raveendran et al. (2019) identified a 25G needle as optimal for maintaining high cell viability during extrusion [79], suggesting a relationship between nozzle geometry and biological performance. Larger nozzle diameters, such as the 580 μm nozzle used by Lee et al. (2023), were associated with thicker filament deposition, larger scaffold dimensions, and potentially coarser scaffold architecture [72]. The consistent reporting of nozzle size across studies indicates recognition of its influence on printed filament diameter, cell damage, and overall scaffold resolution.

#### 3.3.3. Gelation Time and Temperature

Gelation temperature (T_sol-gel_) in biomaterial inks used for 3D bioprinting refers to the temperature at which the material transitions from a liquid (sol) state to a semi-solid or gel state [92]. This property is critical because it determines the ink’s ability to be extruded smoothly through the printing nozzle while maintaining its shape after deposition. An optimal gelation temperature ensures a balance between fluidity during printing and structural stability post-printing, thereby contributing to high print fidelity and accurate layer-by-layer construction [93].

Gelation and printing temperatures varied considerably across studies, reflecting the distinct thermodynamic requirements of different bioink formulations. Gelation temperatures ranged from −20 °C (used by Lai Suo et al., 2022, for thermal crosslinking) [69] to 45 °C (reported by Anitua et al., 2022) [84], with most studies clustering around room temperature (20–25 °C) or at physiological temperature (37 °C). Jiang et al. (2018) specified a gelation temperature of 40 °C for 1 h [68], while Raveendran et al. (2019) utilised a 37 °C water bath for gelation within a temperature-controlled protocol [79]. Notably, several researchers employed multi-stage thermal strategies; Jiang et al. (2020) maintained the barrel at 15 °C and the work stage at 10 °C to stabilise the pore structure and achieve the highest final strut height (15.0 mm) [68]. Similarly, Kim et al. (2022) set the barrel temperature to 10 °C and the printing stage to 38 °C to balance mechanical stability and minimise shear stress [73]. This diversity suggests that optimal temperature profiles are bioink-dependent, with researchers tailoring thermal parameters to modulate viscosity, gelation kinetics, and cell viability simultaneously.

In bioprinting, gelation temperature also plays a key role in preserving cell viability, as many bioinks are cell-laden and must operate under mild, physiologically relevant conditions, typically around 37 °C. For example, temperature-sensitive materials such as gelatine-based bioinks gel upon cooling, usually below approximately 30 °C, which necessitates the use of a cooled print bed to facilitate rapid solidification. In contrast, bioinks such as GelMA rely primarily on photocrosslinking rather than temperature changes, making gelation temperature less influential in their stabilisation. Similarly, alginate-based bioinks gel through ionic crosslinking in the presence of divalent cations such as calcium ions, rather than through thermal mechanisms. Overall, gelation temperature defines the operational window in which a bioink can be effectively printed while retaining its structural integrity. If the gelation temperature is not well optimised, the printed construct may exhibit poor resolution, deformation, or collapse, and in cell-laden systems, it may also compromise cell survival.

#### 3.3.4. Pressure and Flowability

Extrusion pressure varied substantially across studies, ranging from 20 kPa to 0.38 MPa, reflecting differences in bioink viscosity, nozzle geometry, and target deposition rates. Lower pressures (20–50 kPa) were typically used with smaller-volume flow systems, as in Ma et al. (2017) at 50 kPa and Lee et al. (2023) at 20 kPa [66,72]. Medium-range pressures (130–230 kPa) were frequently employed, as demonstrated by Park et al. (2020), who used 130–160 kPa, and by Jeong et al. (2020), who used pressures ranging from 230 to 430 kPa depending on composition (gelatine/tricalcium phosphate ratios) [71,76]. Higher pressures (0.28–0.38 MPa) were used by Jiang et al. (2018) and Tu et al. (2023), likely to accommodate higher-viscosity bioinks or to achieve faster deposition rates [64,68]. Critically, Mohabatpour et al. (2022) demonstrated compositional sensitivity, showing that alginate concentration directly affected the required extrusion pressure, which ranged from 0.2 to 0.7 bar [83]. This relationship highlights the importance of systematically optimising pressure parameters in relation to bioink formulation, as lower-viscosity compositions required adjusted pressure profiles to ensure consistent strand geometry.

#### 3.3.5. Crosslinking Mechanism

Gelation and crosslinking mechanisms were a major point of divergence across studies, reflecting the chemical nature of the hydrogel systems used. Chemical crosslinking via calcium chloride (CaCl_2_) was widely used, particularly for alginate-based hydrogels. Mohabatpour et al. (2022) employed crosslinking in a 50 mM CaCl_2_ solution containing 0.1% polyethyleneimine (PEI), followed by secondary crosslinking in 100 mM CaCl_2_ for 10 min [83]. Similarly, Athirasala et al. (2019) used simultaneous coaxial dispensing of a calcium chloride solution during printing to achieve immediate ionic crosslinking at the nozzle tip [77], whereas Miao et al. (2023) performed post-printing crosslinking in 4% CaCl_2_ for 5 min, followed by UV irradiation at 365 nm for 30 s [78]. Thermal crosslinking was used in alternative systems; Han et al. (2021) crosslinked fibrin-based bioinks with a thrombin solution (10 U/mL) at room temperature for 30–45 min [74]. Photo-initiated crosslinking emerged as a complementary strategy, particularly in studies utilising GelMA and similar photopolymerisable hydrogels. Lin et al. (2021) applied UV light at 405 nm for 90 s, whereas Yang et al. (2023) and Yu et al. (2024) employed UV/DLP systems with controlled light exposure (365–405 nm, 30 s) [67,70,82]. These divergent crosslinking approaches highlight the substrate specificity of gelation protocols and the importance of matching post-printing solidification chemistry to the bioink composition and the target application.

#### 3.3.6. Scaffold Dimensions Specification

Reported scaffold dimensions varied in specificity and target size, ranging from small disc-shaped constructs (5 × 2 mm) to larger cubic or cylindrical scaffolds (30 × 30 × 2 mm or 10 × 10 × 5 mm^3^). Smaller dimensions were often used in cell viability and biocompatibility assays, as exemplified by Athirasala et al. (2018), who produced 5 × 2 mm discs, and by Yu et al. (2024), who created 4 × 4 × 1 mm structures for standardised biological testing [67,77]. Medium-sized scaffolds (5 × 5 × 5 mm^3^ to 10 × 10 × 1.3 mm^3^) were more commonly used for mechanical and biological evaluation, as demonstrated by Han et al. (2021), Raveendran et al. (2019) [1], and Kim et al. (2022) [73,74,79]. Larger constructs intended for more substantial tissue regeneration studies were reported by Lai Suo et al. (2023) with dimensions of 30 × 30 × 2 mm [69]. The specification of layer numbers alongside dimensional parameters revealed standardised approaches. For instance, Raveendran et al. (2019) printed four layers with a fibre spacing of 0.8 mm [79], while Mohabatpour et al. (2022) deposited 31 layers in a 10 × 10 × 5 mm construct [83]. This dimensional variation reflects differing experimental objectives: smaller structures facilitate rapid biological validation, whereas larger constructs enable the study of nutrient diffusion, mechanical properties, and tissue maturation.

#### 3.3.7. Key Similarities and Differences

Despite substantial variation in specific parameters, several core similarities emerged across the reviewed studies. First, all researchers prioritised documenting and controlling the gelation temperature, recognising it as a critical variable that influences bioink viscosity and crosslinking kinetics. Second, nozzle size was universally reported, emphasising its role in determining printed filament dimensions and cell viability. Third, printing pressure was systematised in nearly all studies, indicating standardised approaches to translating pressure into volumetric flow rate. Fourth, post-printing treatment, whether thermal, chemical, or photochemical, was consistently applied across all studies, reflecting universal recognition that extrusion-printed hydrogels require stabilisation before biological or mechanical testing. Finally, dimensional specifications of the final scaffolds, including layer numbers and spacing, were reported in 14 of 21 studies, demonstrating consensus on the need for precise geometric reporting to ensure reproducibility and comparative analysis.

The most substantial differences across studies were strongly correlated with bioink chemistry and crosslinking mechanisms. Alginate-based systems (used by Mohabatpour et al., 2022 and Miao et al., 2023) required rapid ionic crosslinking and typically utilised lower processing temperatures (room temperature to 20 °C) to prevent premature gelation [78,83]. Conversely, protein-based hydrogels, such as gelatine/GelMA composites (used by Raveendran et al., 2019, and Kim et al., 2022), often require physiological or elevated gelation temperatures (37–40 °C) for thermal or photochemical crosslinking [73,79]. Fibrin-based systems (Han et al., 2021) used low printing temperatures (4 °C enclosure) followed by enzymatic crosslinking, a strategy distinct from both ionic and photochemical approaches [74]. Commercial versus custom-built bioprinter systems also influenced parameter reporting; studies using established commercial platforms (BioX, Bio-Architect, Bioplotter) typically reported more complete parameter sets with manufacturer-specified ranges [65,68,79], while custom-built systems sometimes lacked specification of certain parameters (such as gelation time or complete temperature profiles) [66,69]. Additionally, incorporating reinforcement materials such as cellulose nanofibres [68] or tricalcium phosphate [71] required adjusted pressure profiles and printing speeds compared with single-component hydrogel systems.

#### 3.3.8. Clinical and Research Implications

The heterogeneity in reported parameters, despite apparent consensus on key variables, highlights the customised nature of bioprinting protocol development and the bioink-dependent optimisation landscape. For researchers designing new bioprinting studies, this analysis suggests a hierarchical optimisation approach: (1) select the gelation temperature based on the bioink chemistry and the target cell type; (2) determine the nozzle size based on the desired filament diameter and biological requirements; (3) calibrate the extrusion pressure to the bioink viscosity and flow rate; (4) select a crosslinking strategy (ionic, thermal, photochemical, or enzymatic) matched to the bioink composition; and (5) specify the final scaffold dimensions according to the intended application and testing requirements. The widespread adoption of extrusion-based approaches, together with the success of multiple distinct crosslinking strategies, indicates that bioprinting technology has reached sufficient maturity for diverse tissue engineering applications, though continued parameter optimisation remains essential to maximise biological performance and achieve clinical translation.

### 3.4. Physical Assessments of 3D-Bioprinted Hydrogels

3D-bioprinted hydrogels have emerged as promising biomaterials for tissue engineering, with their physical properties critically determining biological performance. A comprehensive analysis of 21 studies reveals substantial diversity in material composition, processing parameters, and resulting physical characteristics. A systematic assessment of swelling behaviour, pore architecture, microstructural organisation, surface morphology, and biodegradation kinetics provides essential insights into the design parameters required for successful tissue regeneration.

The physical assessment of 3D-printed hydrogels encompasses multiple complementary parameters that collectively determine their utility in tissue engineering. The swelling ratio is a critical measure of the hydrogel’s water absorption capacity, which is essential for nutrient exchange and waste removal within the matrix [83]. Pore-size architecture directly influences cell migration, proliferation, and nutrient diffusion, with optimal pore sizes ranging from 100 to 500 μm depending on the application [72]. Microstructural porosity and scanning electron microscopy (SEM) analysis provide visual confirmation of architectural features, while in vitro biodegradation profiles establish the temporal stability of scaffolds during tissue regeneration. The interdependence of these parameters necessitates careful optimisation of formulation design and processing conditions, as shown in Table 3.

#### 3.4.1. Swelling Ratio or Capacity

The swelling ratio of 3D-bioprinted hydrogels is the extent to which a hydrogel absorbs and retains fluid relative to its original (dry or initial) weight or volume [94]. It is a key parameter that quantifies how much a hydrogel expands when placed in an aqueous environment, such as physiological fluids. A higher swelling ratio indicates a greater capacity for the hydrogel to absorb water [94].

In 3D bioprinting, the swelling ratio is a key property because it directly affects the structural stability, porosity, and biological performance of the printed scaffold [95]. Hydrogels with a high swelling ratio typically have larger pore sizes and enhanced nutrient diffusion, which can support cell survival and tissue integration. However, excessive swelling can cause deformation, loss of shape fidelity, or weakened mechanical properties, which is particularly critical in load-bearing applications such as bone or periodontal regeneration. The swelling behaviour of hydrogels depends on several factors, including polymer composition, crosslinking density, and environmental conditions such as pH and temperature [95]. For instance, hydrogels with lower crosslinking density generally exhibit higher swelling ratios due to increased space within the polymer network, whereas highly crosslinked systems restrict water uptake and maintain better mechanical integrity.

Swelling capacity varies markedly across hydrogel compositions, ranging from minimal absorption (0.2%) to exceptional water uptake (1200%), depending on material selection and crosslinking density. The classification of swelling behaviour reveals four distinct categories: low swelling (<15%); moderate swelling (15–50%), observed in alginate and gelatine formulations; high swelling (50–200%), observed in composite systems; and very high swelling (>200%), characteristic of GelMA-based hydrogels.

Material composition emerges as the primary determinant of swelling behaviour, with polysaccharide content and crosslinking density exerting dominant influences. The concentration of carboxymethyl chitosan in alginate–CMC hydrogels directly influences swelling capacity, as a lower crosslinking density allows increased water retention [83]. Mohabatpour et al. demonstrated this relationship across three alginate–CMC compositions on day 21: Alg4–CMC2% achieved the highest swelling ratio of 43.58 ± 0.713, whereas Alg2–CMC4% showed 35.69 ± 1.643, establishing that CMC concentration is inversely correlated with swelling capacity [83]. For instance, Lai Suo et al. documented that CNT-reinforced scaffolds exhibited swelling ratios of 17% in control groups, which decreased to 13% with 0.1% CNT incorporation, demonstrating how additive inclusion can modulate water absorption [69]. In contrast, Ma et al. reported that GelMA/PEGDA hydrogels with volume ratios from 1/4 to 5/0 exhibited swelling ratios ranging from approximately 750% to 1200%, with pure GelMA showing the highest absorption at 1200% [66].

The equilibrium swelling ratio (ESR) quantifies water absorption at saturation, enabling direct comparison across formulations. Tian et al. reported ESR values of 415.7% for S3 bioscaffolds versus 125.9% for S3G7H5 bioscaffolds, highlighting the impact of compositional modifications. In this case, the introduction of gelatine and hydroxyapatite significantly reduces water absorption capacity [65]. High swelling capacity in composite systems arises from the hydrophilic nature of polysaccharide components and the expanded network structure formed during printing and processing. Anitua et al. reported scaffold swelling capacities ranging from 80% to 120% of the initial weight across GA, GAHA, and GAHAP formulations, with GA scaffolds showing the highest 24 h swelling ratio of 12, compared with 9 for GAHA and 10 for GAHAP [84]. These findings highlight that hydroxyapatite incorporation systematically reduces swelling behaviour, likely through increased hydrophobicity and reduced water-binding capacity of the composite matrix. In contrast, in a study by Yang et al. (2023), the swelling ratio of the GelMA hydrogels did not differ significantly with the addition of decellularised ECM [70]. This decellularisation process aimed to create a scaffold that mimics the natural ECM, providing a suitable environment for cell behaviour and tissue regeneration.

The temporal dynamics of swelling indicate that most hydrogels reach equilibrium within 48 h of hydration, although kinetic profiles vary significantly. Tu et al. observed rapid water absorption during the first hour of immersion, followed by a stable swelling plateau of approximately 4.58 after 2 h, indicating a fast saturation kinetic profile [64]. Amoli et al. similarly documented high swelling ratios of 6–8 times, with maximum values within 48 h. However, specific genipin concentrations modulated this behaviour, with 0.5% genipin showing the highest swelling at 8.2, and 2.5% genipin the lowest at 6.8 [81]. The incorporation of BGMs into GelMA/sodium alginate (SA) scaffolds significantly reduced the hydration degree from 85% in GelMA/SA to 80% in GelMA/SA/BGM, illustrating that the incorporation of inorganic particles impairs water absorption capacity [78]. This inverse relationship between additive content and swelling capacity reflects the physical interactions among hydrogel components and their cumulative effect on crosslinking density and network structure.

#### 3.4.2. Pore Size

Pore size analysis across the literature reveals a remarkably broad range, spanning 4 to 800 micrometres, with a mean of 226.7 μm and a median of 100 μm, reflecting the diversity of tissue engineering applications and processing parameters. The smallest pore sizes arise from advanced printing techniques. Messaoud et al. reported pore sizes of 4–100 μm for interconnected porous (ICP) hydrogels, compared with 15–70 μm for regular disconnected porous (RDP) variants [80]. Mid-range pore sizes (200–500 μm) are most commonly reported in the literature, reflecting optimisation for cell migration and nutrient diffusion. Kim et al. documented 500 μm pores in gelatine-based constructs, while Tu et al. reported approximately 600 μm pore dimensions [64,73]. The largest pore sizes (>600 μm) occur in alginate-based systems designed for macroporous tissue engineering. Mohabatpour et al. reported pore sizes of 716 ± 0.054 mm in Alg2–CMC4% compositions, which represent the upper limit for scaffold applications [83].

Pore size tunability via processing parameters is critical for application-specific design. Jiang et al. showed that pore size can be systematically reduced from 400 μm (designed) to 200 μm by varying the barrel temperature during extrusion printing, with specific temperatures producing targeted pore diameters: 10 °C for an initial strut height of 4 mm, 15 °C for 5 mm, 20 °C for 3 mm, and 25 °C for 2 mm [68]. In a study by Ma et al. (2017), the inverse correlation between GelMA content and pore size in GelMA/PEGDA composites provides additional evidence of compositional control, with pore sizes increasing from 55.7 ± 10.1 μm at the 1/4 GelMA/PEGDA ratio to 248.9 ± 35.5 μm at the 5/0 ratio [66]. Likewise, genipin concentration in crosslinked gelatine hydrogels shows an inverse relationship with pore size, with 0.5 wt% genipin producing larger pores (86.3 ± 8.1 μm) than 2.5 wt% genipin (63.6 ± 6.8 μm) [81], confirming that crosslinking density is a fundamental determinant of microarchitecture.

#### 3.4.3. Microstructure Porosity

The architecture of pore networks critically determines nutrient diffusion and cell behaviour, with interconnected porous structures emerging as optimal for tissue engineering applications. Jeong et al. described an interconnected porous microstructure with a uniform distribution of β-TCP nanoparticles and controlled architectural features, including a 500 μm strand thickness and 700 μm strand spacing [71]. Maintaining well-defined intersections in 3D-bioprinted structures enables systematic nutrient transport and waste removal. Mohabatpour et al. observed a “highly porous structure with well-defined intersections” in their Alg-CMC scaffolds. Their key finding was that scaffolds with higher carboxymethyl chitosan ratios exhibited smaller filament sizes while maintaining superior pore morphology [83]. Fibre dimension standardisation across studies typically ranges from 300 to 500 μm spacing, with Lai Suo et al. documenting fibres of approximately 500 μm dimension and 300–400 μm spacing [69].

#### 3.4.4. Morphology

Scanning electron microscopy analysis reveals four primary surface characteristics across the 3D-bioprinted hydrogel literature: porous structures, observed in nine papers [62,64,66,67,68,70,76,79,82]; rough surfaces in four papers [63,69,73,76]; smooth surfaces in three papers [63,66,76]; and fibrillar features in one paper [78]. The prevalence of porous structures highlights the fundamental need for open-network architectures in tissue engineering applications. Messaoud et al. reported that GelMA hydrogels exhibited fibrillar features, RDP hydrogels showed isolated, disconnected pores, and ICP hydrogels displayed a nanofibrous architecture, establishing that material formulation and processing parameters directly determine surface morphology [80]. The incorporation of nanoparticles and bioactive components systematically modifies surface characteristics and microstructural organisation. The importance of surface roughness for cell attachment is illustrated by Jeong et al.’s observation that β-TCP nanoparticles created a rough surface, providing a suitable environment for cell attachment, with preosteoblasts extending filopodia on this roughened surface [71]. The study by Lai Suo et al. demonstrates that CNTs produce loose, porous structures that promote the proliferation of periodontal ligament cells through their rough surface and internal pore architecture [69].

The development of hierarchical surface architectures through material incorporation significantly improves biological functionality. Miao et al. reported that GelMA/SA/BGM scaffolds exhibited numerous small pores within their filaments and rough surfaces, resulting from the uniform encapsulation of bioactive glass microspheres. This contrasts markedly with the relatively smooth and intact filament structure observed in GelMA/SA scaffolds [78]. Dubey et al. observed that AMP powder, consisting of micron-sized particles with plate-like structures, produced rougher bioink surfaces (ECM/AMP) than ECM alone, which has a tight, dense, and wrinkled surface [75].

#### 3.4.5. In Vitro Biodegradation

Biodegradation kinetics show substantial variation across hydrogel compositions, with degradation rates grouped into three categories: rapid (>30% mass loss), moderate (15–30% mass loss), and slow (<15% mass loss). Rapid degradation, observed in approximately six studies (46% of papers reporting degradation), characterises gelatine-only and highly GelMA-enriched hydrogels [66,69,70,76,78,81]. Ma et al. reported that pure GelMA hydrogels (5/0 ratio) degraded almost completely by day 7, which was considered too rapid for effective tissue regeneration, demonstrating the necessity of composite formulations to achieve physiologically appropriate degradation timescales [66]. Lai Suo et al. observed degradation rates of 35.69 ± 2.46% on day 21 in control chitosan–alginate–alginate laurate (CS/AL) groups, with CNT incorporation systematically slowing degradation to 21.15 ± 2.89% at a 1% CNT concentration [69]. Amoli et al. demonstrated that genipin-crosslinked gelatine scaffolds achieved maximum degradation within 14 days of immersion in PBS. Degradation was highest at 0.5% genipin, reaching 41%, and decreased to 20% at 2.5% genipin concentration [81]. Park et al. found that both GelMA and BMP-GelMA hydrogel constructs experienced about 60% mass loss by day 28 [76].

Moderate degradation rates (15–30%), characterised in four studies, are considered optimal profiles for many tissue engineering applications [64,67,71,82]. Jeong et al. reported that gelatine-only scaffolds degraded by 16% by day 28, which was the fastest degradation among their tested formulations [71]. Lin et al. observed controlled degradation in various chitosan (CS) compositions: CS0 with 12% weight loss, CS5 with 18%, and CS10 with 24%. They found that increased crosslinking in chitosan systematically raises the degradation rate [82]. Tu et al. reported 17% degradation after 16 weeks in simulated body fluid, with structural integrity maintained for at least 4 weeks, whereas Yu et al. documented approximately 20% degradation after 15 days in PBS [64].

Slow degradation rates (<15%), representing the most stable formulations, are reported in one study [84]. In a study by Anitua et al., the GAHAP gels exhibited the lowest degradation rate among the tested scaffolds. This indicates that they maintained their structure better over time than other formulations, such as the gelatine and alginate scaffolds (GA), which degraded more quickly under simulated physiological conditions [84]. The temporal degradation patterns reveal critical compositional effects that enable fine-tuning of scaffold longevity. The incorporation of β-TCP nanoparticles substantially reduces gelatine degradation rates. Jeong et al.’s findings show that gelatine-only scaffolds degraded by 16%, whereas those with 20% β-TCP incorporation (G80T20) degraded by only 4%, establishing that mineral content inversely correlates with enzymatic susceptibility [71]. The GelMA/PEGDA composite system exhibits composition-dependent degradation, with increased PEGDA incorporation significantly slowing degradation. Hydrogels with ratios from 4/1 to 1/4 showed significantly slower degradation rates, with mass remaining even after 14 days, which is in sharp contrast to the degradation of pure GelMA [66]. Similarly, incorporating bioactive glass microspheres (BGMs) enhances stability. Miao et al. reported that after 48 h, GelMA/SA degradation left 21.42% of the mass remaining, whereas GelMA/SA/BGM maintained 37.12% mass retention due to reduced water absorption, which limited adsorption of the collagenase solution [78].

The relationship between pore size and degradation rate provides further mechanistic insight into temporal dynamics. Larger pore sizes facilitate faster enzyme diffusion and substrate access, resulting in accelerated degradation kinetics. In Ma et al., the 4/1 GelMA/PEGDA ratio, which has the largest pore size among all the ratios, exhibits the highest degradation rate [66]. The systematic investigation of these composition-degradation relationships shows that controlled degradation can be engineered through material selection, enabling designers to match scaffold longevity to specific tissue regeneration timescales, from rapid (7 days) for applications requiring host–tissue integration to slow (>16 weeks) for sustained structural support.

### 3.5. Rheological Assessments of 3D-Bioprinted Hydrogels

The successful development of 3D-bioprinted constructs depends critically on understanding and optimising the rheological properties of hydrogel bioinks. Rheological characterisation measures how materials deform and flow under applied stress, directly determining a hydrogel’s suitability for extrusion-based three-dimensional printing. The relationships between viscosity, shear rate, storage modulus (G′), and loss modulus (G″) provide essential information for predicting printability, structural integrity, and the ability to support tissue regeneration [96]. These properties must be carefully balanced to ensure both adequate extrusion through the printer nozzle and sufficient mechanical strength in the printed construct. The challenge in bioink design lies in formulating materials that exhibit favourable flow characteristics during printing while maintaining structural stability and mechanical competency post-printing to support cellular activities [97,98].

Multiple studies have demonstrated that hydrogel rheological properties are influenced by polymer composition, concentration, the addition of fillers or a crosslinking agent, and environmental factors, as shown in Table 4. The storage of mechanical energy (G′) relative to its dissipation (G″) is a primary indicator of whether a material behaves predominantly as an elastic solid or a viscous fluid. For 3D-bioprinting applications, a higher storage modulus compared to loss modulus is essential, as it indicates that the material can maintain its shape and structural integrity after extrusion [99]. Understanding these fundamental relationships enables researchers and clinicians to design optimised bioinks tailored to specific tissue engineering applications, whether for periodontal regeneration, cartilage engineering, or other complex tissue structures.

#### 3.5.1. Shear Rate, Shear Strength, and Viscosity

Shear-thinning behaviour represents one of the most critical rheological properties for 3D-bioprinting applications [100]. When the viscosity of a material decreases as the shear rate increases, the material is classified as a shear-thinning or pseudoplastic fluid [101]. This behaviour is universally beneficial for extrusion-based 3D-bioprinting, as it allows bioinks to flow more easily through the printer’s narrow nozzle at high shear rates while recovering their gel-like properties immediately upon exiting the nozzle [102]. Jeong and colleagues demonstrated that hydrogels evaluated across shear rates ranging from 0 to 100 s^−1^ exhibited consistent shear-thinning behaviour, confirming that these materials were extrudable and suitable for printing [71]. Similarly, studies examining viscosity at varying shear rates confirmed that as the shear rate increased, the material viscosity decreased, allowing for easier extrusion through the printer nozzle without requiring excessive pressure [64,65,68,70,74,75,79].

The practical implications of shear-thinning behaviour for clinical and research applications cannot be overstated. Materials that maintain high viscosity at rest (zero shear rate) but thin dramatically during extrusion (high shear rate) provide optimal printability while minimising the risk of diffusion or loss of structural definition during printing. This behaviour enables the formulation of bioinks with enhanced structural integrity, which would otherwise require excessively high nozzle pressures. Research by Amoli and colleagues emphasised that shear-thinning enables materials to flow under high-shear conditions, making them particularly suitable for extrusion-based three-dimensional bioprinting applications, where resolution and precision are paramount [81].

**Figure 7:**
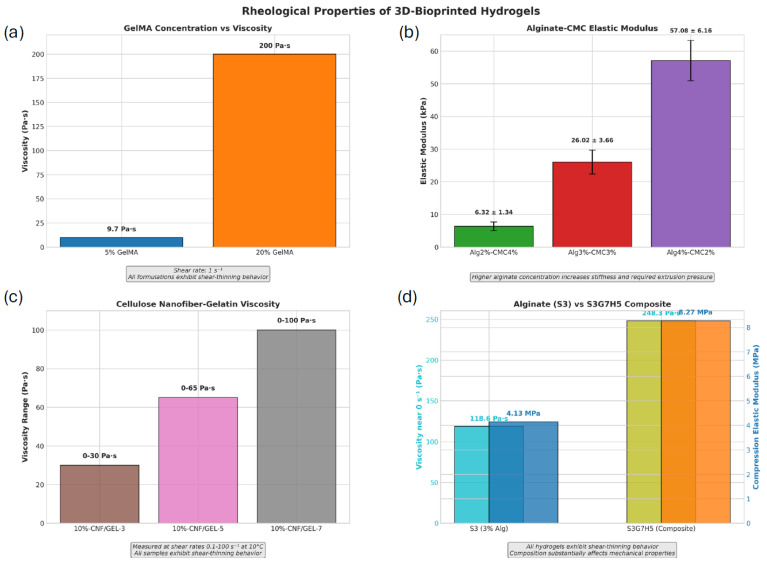
Image showing viscosity comparisons across different material compositions. (**a**) GelMA exhibits concentration-dependent viscosity behaviour, with 5% (*w*/*v*) GelMA showing approximately 9.7 Pa·s at 1 s^−1^, while the same measurement for 20% (*w*/*v*) GelMA yields a viscosity of 200 Pa·s [79]. (**b**) The comparison also shows that Alg2–CMC4% combinations have the lowest viscosity among the tested groups, requiring the lowest extrusion pressure, whereas solutions with higher alginate concentrations (Alg3–CMC3% and Alg4–CMC2%) have significantly higher viscosities and require higher extrusion pressures [83]. (**c**) Viscosity increases with higher gelatine content in the CNF–gelatine formulations. All samples exhibited shear-thinning behaviour, with viscosity decreasing as the shear rate increased, which is beneficial for 3D printing applications. The progressive increase in viscosity from 10–CNF/GEL-3 to 10–CNF/GEL-7 reflects how gelatine incorporation can be tuned to achieve the desired flow properties while maintaining the structural benefits of cellulose nanofibre reinforcement [68]. (**d**) Additionally, for materials such as S3 and S3G7H5 hydrogels, shear viscosity testing at shear rates near 0 s^−1^ revealed maximum viscosities of 118.6 and 248.3 Pa·s, respectively. Both materials were classified as shear-thinning, non-Newtonian fluids [65]. According to Tian et al., the ideal viscosity for 3D bioscaffold printing is generally recommended to be below 1000 Pa·s, with an optimal range of 30 to 300 Pa·s [65].

The magnitude of viscosity and shear-thinning behaviour varies significantly with material composition and concentration. A study by Raveendran et al. on GelMA at different concentrations revealed that a 5% (*w*/*v*) GelMA formulation exhibited a viscosity of approximately 9.7 Pa·s at 1 s^−1^, whereas the same measurement for 20% (*w*/*v*) GelMA yielded a viscosity of 200 Pa·s, as shown in Figure 7a [79]. Despite substantial differences in absolute viscosity, both formulations exhibited shear-thinning behaviour, with viscosity decreasing gradually as the shear rate increased, indicating suitable extrusion characteristics across a broad concentration range. The viscosity of all hydrogel precursors decreased gradually with increasing shear rate, indicating flow behaviour under high shear conditions that made the hydrogel precursors suitable for extrusion [79].

When examining alginate-based formulations, researchers identified even more pronounced effects of composition on viscosity. The Alg2–CMC4% combination exhibited the lowest viscosity among the tested groups, requiring the lowest extrusion pressure, whereas solutions with higher alginate concentrations (Alg3–CMC3% and Alg4–CMC2%) showed significantly higher viscosities and required higher extrusion pressures, as shown in Figure 7b [83]. These concentration-dependent viscosity variations demonstrate that formulators can fine-tune extrusion characteristics by adjusting polymer composition, creating a spectrum of bioinks ranging from low-viscosity, easily extrudable formulations to high-viscosity options with enhanced mechanical properties. In a study by Han et al., the DDMp bioink showed that viscosity increases with particle concentration, and higher concentrations yield greater viscosity, thereby enhancing both printability and the structural integrity of the printed constructs [74].

Different studies employed different shear-rate ranges during rheological characterisation, yet the observed shear-thinning behaviour was consistently reported across these diverse measurement protocols. The most commonly tested range spanned 0.1 to 100 s^−1^, approximating the range encountered during actual extrusion printing [68,70,74,77]. Messaoud and colleagues tested shear rates from 10^−2^ to 10^3^ s^−1^, providing a broader range of characterisation, whereas other researchers focused on narrower windows, from 0.1 to 100 s^−1^ or even 0 to 100 s^−1^ [80]. For materials such as S3 and S3G7H5 hydrogels, shear viscosity testing at shear rates near 0 s^−1^ revealed maximum viscosities of 118.6 and 248.3 Pa·s, respectively, as shown in Figure 7d. Both materials were classified as shear-thinning, non-Newtonian fluids [65]. These maximum viscosities at near-zero shear rates indicate that materials maintain substantial body at rest, which is essential to prevent the collapse or spreading of printed features [65].

#### 3.5.2. Storage (G′) and Elastic (G″) Modulus

The rheological characterisation of hydrogel bioinks evaluates how materials deform and flow under applied stress, with implications for printability, structural stability, and the ability to support tissue regeneration. In 3D bioprinting applications, a higher storage modulus than loss modulus is generally desirable, as it indicates that the material behaves more as a solid-like viscoelastic structure.

**Figure 8:**
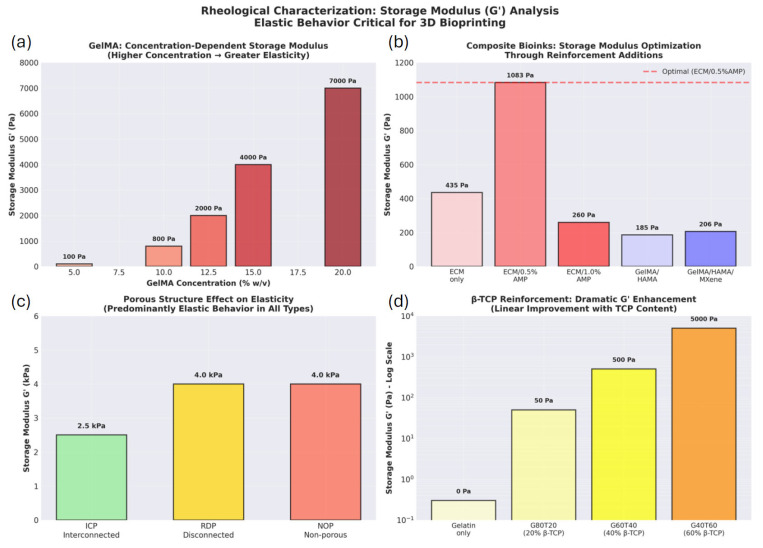
The analysis of G′ reveals its importance in 3D bioprinting. (**a**) GelMA exhibits concentration-dependent effects: Higher concentrations increase viscosity and G′, indicating more solid-like behaviour and stronger mechanical integrity, both of which are crucial for maintaining shape after extrusion. This allows tuning of GelMA bioinks for various tissue engineering needs [79]. (**b**) ECM hydrogel exhibits shear-thinning behaviour, and adding ECM/0.5AMP increases viscosity, with G′ at 1083 Pa, indicating strong shape retention during printing, compared with lower values for ECM alone and ECM/1.0AMP [75]. (**c**) Porous structure effect on elasticity [80]. (**d**) β-TCP reinforcement significantly boosts G′ at 10 Hz: G40T60 reached 5000 Pa, G60T40 500 Pa, and G80T20 50 Pa, and pure gelatine only reached 0.3 Pa, demonstrating a linear enhancement effect of ceramic reinforcement [71].

For bone tissue engineering applications, the ECM/0.5AMP bioink formulation demonstrated optimal viscoelastic performance, with a storage modulus of 1083 Pa at 1 rad s-1, indicating strong viscoelastic gel behaviour capable of maintaining its shape and structural integrity during and after the printing process, compared with 435 Pa for ECM alone and 260 Pa for ECM/1.0AMP, as shown in Figure 8b [75]. In periodontal applications, the 5% (*w*/*v*) GelMA formulation exhibited the highest storage modulus of 1000 Pa and viscosity of 7000 Pa·s, while a G″ of 300 Pa was also reported, as shown in Figure 8a [79].

Despite these promising findings, significant limitations remain in the interpretation and application of the G′ and G″ values. First, only a limited number of studies included detailed reporting of rheological parameters such as storage modulus, loss modulus, and viscosity under standardised conditions, thereby restricting meaningful comparison between studies. Moreover, the rheological assessment methods varied considerably across the included studies, as mentioned above, with some employing shear-rate ranges from 0.1 to 100 s^−1^ [70,71,74,77], while others used broader ranges from 10^−2^ to 10^4^ s^−1^ [65,80,82], complicating direct interpretation. Therefore, the establishment of universally accepted and standardised testing protocols is strongly recommended to improve reproducibility, facilitate meaningful cross-study comparisons, and accelerate regulatory approval and clinical translation of 3D-bioprinted hydrogel scaffolds for dental applications.

Although the included studies extensively characterised the physical and mechanical properties of these hydrogels in vitro, the translation of these properties into predictable clinical outcomes remains insufficiently understood. This gap limits the ability to determine which specific storage and loss modulus values optimally support periodontal, bone, or dental pulp regeneration in real-world clinical settings. Furthermore, most currently available in vivo studies involve relatively short observation periods and may not adequately replicate the complex biological and biomechanical environment of the oral cavity [70,78,103,104,105]. Consequently, greater emphasis should be placed on long-term in vivo validation studies to evaluate scaffold durability, degradation behaviour, tissue integration, and regenerative stability over extended periods.

### 3.6. Mechanical Assessments of 3D-Bioprinted Hydrogels

Mechanical assessment is fundamental to evaluating the performance and clinical applicability of 3D-printed hydrogel scaffolds, particularly in tissue engineering and regenerative medicine. Key mechanical properties, including compressive strength, Young’s modulus, stiffness, and tensile resilience, are commonly measured to assess the scaffold’s ability to withstand physiological loading while maintaining structural integrity. As shown in Table 5, these properties vary widely with bioink composition, crosslinking strategy, and the incorporation of reinforcing agents such as bioceramics, nanofibres, or synthetic polymers. Notably, reinforcement strategies, such as the addition of β-tricalcium phosphate, cellulose nanofibres, or hybrid polymer systems, have been shown to significantly enhance mechanical performance, enabling the fabrication of scaffolds with tunable properties for specific tissue applications.

Mechanical assessment of 3D-bioprinted hydrogels must account for specific tissue requirements to ensure appropriate scaffold design. For instance, cancellous bone tissue, which serves as a reference standard for many bone tissue engineering applications, exhibits a compressive strength of 2–20 MPa and a Young’s modulus of 2–12 GPa [64,71]. Even applications in periodontal tissue regeneration benefit from intermediate mechanical properties that withstand functional loading while remaining compatible with the mechanics of the periodontal ligament. The mechanical properties of scaffolds can be optimised for specific tooth structures, bone augmentation, or periodontal applications.

The role of mechanical properties in directing cellular responses and tissue formation cannot be overlooked in clinical implementation. Overall, understanding and optimising these mechanical characteristics is essential for designing hydrogels that achieve an appropriate balance between mechanical stability and biological functionality in 3D bioprinting applications.

#### 3.6.1. Compressive Strength

Compressive strength is one of the most critical mechanical properties of 3D-bioprinted hydrogels, particularly for applications requiring tissue regeneration and structural support. The compressive strength of hydrogels varies considerably with their composition and reinforcement strategy [106]. Studies have shown that load failure typically occurs at strains of 0.5 to 0.7, with maximum rupture stresses ranging from 15 to 100 kPa for basic formulations [80]. However, when reinforced with bioactive fillers, these values can increase significantly.

**Figure 9:**
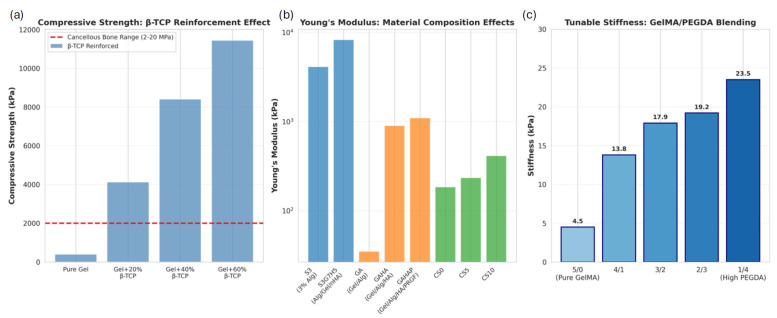
The figure presents three key comparative analyses: (**a**) The effect of β-TCP reinforcement: pure gelatine scaffolds exhibited the lowest compressive strength at 0.4 ± 0.14 MPa, while formulations with increasing β-TCP content showed a progressive increase: G80T20 (20% β-TCP) reached 4.12 ± 0.22 MPa, G60T40 (40% β-TCP) achieved 8.41 ± 1.41 MPa, and G40T60 (60% β-TCP) attained 11.45 ± 1.96 MPa, demonstrating a linear relationship between reinforcement content and mechanical strength. [71]. (**b**) Young’s modulus comparison: S3G7H5 showed a higher Young’s modulus of 8.27 MPa than S3 at 4.13 MPa [65]. Scaffolds with 5% calcium silicate (CS5) reached 234.4 ± 8.2 kPa, with the maximum enhancement at 10% CS (CS10) achieving 413.3 ± 12.5 kPa [83], while hydroxyapatite-reinforced scaffolds exhibited exceptional stiffness, with GAHA formulations achieving a Young’s modulus of 900 kPa and GAHAP reaching 1100 kPa [84]. (**c**) Tunable stiffness (GelMA/PEGDA): Pure GelMA (5/0 ratio) yielded the lowest stiffness at 4.5 ± 2.3 kPa, while systematically decreasing the GelMA-to-PEGDA ratio progressively increased stiffness: 4:1 (13.8 ± 1.7 kPa), 3:2 (17.9 ± 2.3 kPa), 2:3 (19.2 ± 2.1 kPa), and 1:4 (23.5 ± 2.6 kPa), enabling researchers to tailor scaffolds to specific tissue requirements [66].

Research incorporating β-TCP (beta-tricalcium phosphate) as a reinforcement demonstrated substantial improvements in compressive strength [71]. As shown in Figure 9a, pure gelatine scaffolds exhibited the lowest compressive strength at 0.4 ± 0.14 MPa, while formulations with increasing β-TCP content demonstrated progressive enhancement: G80T20 (20% β-TCP) reached 4.12 ± 0.22 MPa, G60T40 (40% β-TCP) achieved 8.41 ± 1.41 MPa, and G40T60 (60% β-TCP) attained 11.45 ± 1.96 MPa [71]. This linear relationship between reinforcement content and mechanical strength demonstrates the effectiveness of ceramic fillers in enhancing scaffold rigidity. The scaffolds incorporating β-TCP reinforcement exhibited compressive strengths within the range of cancellous bone, indicating their potential to support newly formed bone tissue [71]. However, most soft hydrogels have substantially lower moduli than bone, which reduces stress shielding and provides more suitable stress conditions for tissue regeneration [64].

**Figure 10:**
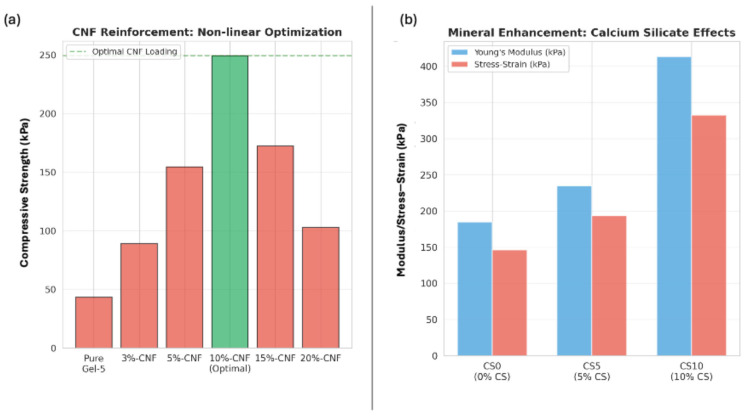
The figure displays other reinforcement strategies to enhance the mechanical performance of scaffolds: (**a**) CNF reinforcement optimisation: Incorporating cellulose nanofibres (CNFs) into gelatine scaffolds produced optimal reinforcement at 10% CNF content, yielding a maximum compressive strength of 249.37 kPa, with data revealing a non-linear response from pure GEL-5 at 43.32 kPa to the peak at 10–CNF/GEL-5 (249.37 kPa), and then declining to 172.47 kPa at 15–CNF, suggesting that excessive nanofibre loading may compromise mechanical properties [68]. (**b**) Calcium silicate enhancement: Dual comparison showing Young’s modulus and stress–strain curves with systematic increases demonstrating how mineral components significantly enhance mechanical performance [83].

Nanofibre reinforcement has emerged as another promising strategy to improve compressive strength. Incorporating cellulose nanofibres (CNFs) into gelatine scaffolds produced optimal reinforcement at 10% CNF content, yielding a maximum compressive strength of 249.37 kPa [68]. The data reveal a non-linear response, with pure GEL-5 at 43.32 kPa, increasing to 89.0 kPa with 3–CNF addition, reaching a peak at 10–CNF/GEL-5 (249.37 kPa), and then declining to 172.47 kPa at 15–CNF and 102.71 kPa at 20–CNF, as shown in Figure 10a [68]. This suggests that excessive nanofibre loading may compromise mechanical properties or disrupt hydrogel network formation. Additionally, carbon nanotube (CNT) reinforcement has shown significant promise, with the 1% CNT/CS/AL group exhibiting the highest compressive strength across the tested formulations, making it particularly suitable for supporting periodontal tissue regeneration [69].

The incorporation of other bioactive materials has also enhanced compressive strength. Polycaprolactone hybrid constructs demonstrated substantially higher strength than pure bioinks, with the PCL-DDMP bioink reaching 30,000 kPa compared with 25,000 kPa for PCL alone [74]. Collectively, these findings indicate that strategic material selection and optimisation of reinforcement content are essential to achieving the desired mechanical performance in 3D-bioprinted hydrogel scaffolds.

#### 3.6.2. Young’s Modulus

Young’s modulus quantifies a material’s resistance to elastic deformation and is a fundamental measure of scaffold stiffness. Characterisation of Young’s modulus across hydrogel formulations shows substantial variation depending on composition and crosslinking strategies. Basic alginate-based formulations showed relatively modest moduli, with pure NOP at 9.0 ± 1.4 kPa, RDP at 13.0 ± 1.5 kPa, and ICP at 9.1 ± 1.5 kPa [80]. These low values highlight the inherent flexibility of natural polymer-based hydrogels, which may be advantageous for certain tissue engineering applications but limiting in load-bearing contexts.

Polymer blending has proven highly effective in modulating mechanical properties. The GelMA/PEGDA system exemplifies this approach, with varying volume ratios dramatically altering material stiffness [66]. Pure GelMA (5/0 ratio) yielded the lowest stiffness at 4.5 ± 2.3 kPa, while systematically decreasing the GelMA-to-PEGDA ratio progressively increased stiffness: 4:1 (13.8 ± 1.7 kPa), 3:2 (17.9 ± 2.3 kPa), 2:3 (19.2 ± 2.1 kPa), and finally 1:4 (23.5 ± 2.6 kPa) [66]. This tunable stiffness approach enables researchers to tailor scaffolds to the specific mechanical requirements of each tissue type.

In a study by Lin et al., incorporating calcium silicate (CS) as a reinforcement phase significantly increased Young’s modulus in CS-containing GelMA scaffolds [82]. Scaffolds without CS (C0) exhibited a modulus of 184.9 ± 6.1 kPa, whereas those with 5% CS (C5) reached 234.4 ± 8.2 kPa. The maximum enhancement occurred at 10% CS (C10), with a modulus of 413.3 ± 12.5 kPa [82]. The presence of mineral components significantly increases stiffness, which is directly correlated with improved compressive strength and greater potential for bone tissue regeneration. Similarly, hydroxyapatite-reinforced scaffolds exhibited exceptional stiffness, with GAHA formulations achieving a Young’s modulus of 900 kPa and GAHAP reaching 1100 kPa, substantially exceeding that of their non-mineralised counterparts [84].

Advanced composites incorporating bioceramics exhibited comparable enhancement patterns. In a study by Tian et al., the composite hydrogel bioscaffolds S3 and S3G7H5 exhibited marked differences in Young’s modulus, with S3 achieving 4.13 MPa and S3G7H5 reaching 8.27 MPa. This indicates that compositional variation substantially affects compressive behaviour, as shown in Figure 9b [65].

#### 3.6.3. Stiffness

Scaffold stiffness, often quantified by compressive or storage modulus measurements, reflects the material’s resistance to deformation under applied force [107]. GelMA-based hydrogels have emerged as popular candidates for 3D printing applications due to their tunable mechanical properties. Engineered GelMA hydrogels exhibited stiffness values of 7.4–14.2 kPa, which is significantly higher than previously reported values for UV-crosslinked GelMA hydrogels at comparable concentrations [80]. This improvement may reflect optimisation of crosslinking strategies or enhanced material formulations.

The characterisation of stiffness via the G′ provides insight into a material’s viscoelastic behaviour. In a study by Dubey et al., the ECM/0.5AMP bioink formulation exhibited the highest stiffness among the tested compositions, with a storage modulus of 1083 Pa, compared with 435 Pa for ECM alone and 260 Pa for ECM/1.0AMP [75]. These findings demonstrate that optimised concentrations of AMP can enhance viscoelastic properties, which are critical for printability and post-print structural integrity.

Alginate-based formulations with carboxymethyl chitosan additions showed variable stiffness depending on the concentration ratios. The Alg4–CMC2% scaffold exhibited the highest elastic modulus of 57.08 ± 6.162 kPa among the tested alginate compositions, indicating superior stiffness [83]. In contrast, Alg2–CMC4% produced much lower values of 6.320 ± 1.339 kPa [83]. The findings show that stiffness can be precisely tuned by adjusting component concentration, enabling the development of scaffolds with application-specific mechanical properties.

Moreover, photocrosslinked 3D-bioprinted scaffolds using 12.5% GelMA exhibited sufficient physical integrity, indicating adequate stiffness for handling with surgical instruments without deformation [79]. Collectively, these studies indicate that assessing stiffness is essential for predicting scaffold behaviour during implantation and integration with host tissues.

#### 3.6.4. Tensile Resilience

Tensile resilience, the material’s capacity to withstand tension without permanent deformation, is an important mechanical property for hydrogel scaffolds subjected to dynamic loading, as hydrogels must endure applied forces while deforming and recovering their original shape under mechanical stress [108]. Several hydrogel formulations demonstrated remarkable resilience, with scaffolds capable of being compressed to at least 60% of their original width while still fully recovering their original form [64]. This elastic behaviour is critical for scaffolds intended to support tissues subjected to cyclic mechanical stress, such as cartilage or the periodontal ligament.

The addition of reinforcing fibres substantially enhanced tensile resilience. When cellulose nanofibres were incorporated into gelatine scaffolds, the maximum deformation of 10–CNF/GEL-5 exceeded that of pure GEL-5, indicating improved tensile resilience [68]. The stress–strain curves showed that formulations with higher nanofibre content exhibited steeper curves, indicating a greater capacity to withstand higher strain than unmodified samples [68]. This suggests that nanofibre reinforcement not only increases material stiffness but also improves elasticity and recovery properties.

In a study by Lin et al., the incorporation of calcium silicate similarly enhanced tensile properties [82]. Scaffolds with increased CS content exhibited steeper stress–strain curves, indicating improved capacity to withstand higher strain, enabling better manipulation during surgical procedures and better adaptation to native pressures, as shown in Figure 10b [82]. The presence of covalent bonds provided by CS appeared to progressively increase the stress–strain relationship as CS content increased [82]. Such enhancements are particularly valuable in tissue engineering, where scaffolds must withstand physiological loading while maintaining dimensional stability.

Bioink composition strategies have been specifically designed to enhance tensile resilience. The combination of GelMA and HAMA with MXene in bioinks aims to mimic the ECM, providing a supportive environment that can withstand tensile forces while promoting cell growth and differentiation [72]. Storage modulus measurements before and after UV crosslinking showed significant increases, rising from approximately 2 kPa to 4 kPa after crosslinking, indicating that processing conditions substantially influence material elasticity [76]. While the G′ value and tensile resilience are not the same property, a higher G′ value generally indicates a stiffer material that stores more energy during deformation, which is a key component of high resilience. These findings establish that tensile resilience is a multifactorial property, influenced by material composition, reinforcement strategy, and crosslinking approach.

## 4. Overview of 3D-Bioprinted Hydrogels for Dental Applications

The development of 3D-bioprinted hydrogels for dental applications requires selecting biomaterials tailored to the specific requirements of different dental tissues, taking into account their physical, mechanical, and rheological properties. The research highlights distinct material compositions and reinforcement strategies that enable the customisation of scaffolds for diverse regenerative applications, including bone, periodontal, dentine, pulp, and soft tissues.

In bone tissue regeneration, gelatine-based hydrogels, particularly those reinforced with β-TCP, have shown considerable promise [35]. Research indicates that pure gelatine scaffolds have low compressive strength; however, as β-TCP content increases, compressive strength rises significantly, reaching levels comparable to those of cancellous bone [71]. For instance, formulations containing 60% β-TCP achieved a value of 11.45 ± 1.96 MPa, making them suitable for supporting newly formed bone tissue. Additionally, materials such as GelMA combined with PEGDA [66] or HAMA [72] have been investigated, with stiffness adjustable by varying polymer ratios. Moreover, composites comprising sodium alginate, gelatine, and nano-hydroxyapatite exhibited improved Young’s modulus, indicating their potential suitability for load-bearing bone applications [64,65]. The morphology of these scaffolds, characterised by rough surfaces generated by nanoparticles, also creates an environment conducive to cell attachment and proliferation [65,71].

In periodontal tissue regeneration, hydrogels are engineered to support both soft and hard tissues. GelMA is a prominent biomaterial, often photocrosslinked to enhance structural integrity and printing fidelity [78,79]. GelMA-based hydrogels can be combined with other polymers, such as sodium alginate (SA) and bioactive glass microspheres (BGMs), to improve mechanical properties and degradation rates [78]. Chitosan, sodium alginate, and CNT composites have also shown promise, with CNT incorporation enhancing compressive strength, making them suitable for periodontal applications [69]. The ability of these hydrogels to maintain specific pore sizes (e.g., 400 μm for cell growth) [69] and their moderate degradation rates are crucial for facilitating cell migration and tissue remodelling in the periodontal ligament [69,78].

For dentine and pulp regeneration, biomaterials must support dental pulp stem cells and promote odontogenic differentiation. DDM incorporated into fibrinogen–gelatine mixtures has been used, with DDM particle concentration influencing viscosity and structural integrity [74]. Calcium silicate in GelMA hydrogels has been shown to increase Young’s modulus and stiffness, both of which are crucial for dentine-like structures [82]. GA hydrogels, particularly when enriched with hydroxyapatite or with hydroxyapatite and plasma rich in growth factors, exhibit increased stiffness, with GAHAP reaching a Young’s modulus of 1100 kPa [84]. These materials often exhibit controlled degradation rates and highly porous structures that are beneficial for cell survival and nutrient exchange within the complex dentine–pulp environment [84].

Enamel regeneration has been explored using composites of CMC and Alg [83]. These dual-crosslinked hydrogels can form multilayered structures with well-defined pores. Studies have shown that HAT-7 cells retain normal morphology on these scaffolds, indicating biocompatibility. Mineral deposition observed in these scaffolds suggests their potential to promote ameloblast differentiation and enamel mineralisation [83].

For soft tissues in the dentoalveolar region, maleic anhydride-grafted chitosan and gelatine hydrogels crosslinked with genipin have been investigated [81]. These hydrogels exhibit tunable swelling ratios, pore diameters, and degradation rates that depend on genipin concentration, enabling customisation to soft-tissue requirements. Their high porosity and interconnected networks facilitate cell infiltration and nutrient exchange, which are both essential for soft-tissue healing and integration [81].

## 5. Strengths and Limitations

This scoping review demonstrates several significant strengths in its comprehensive examination of 3D-bioprinted hydrogel scaffolds for dental tissue engineering. First, the review systematically characterises physical and mechanical properties across multiple assessment categories, including swelling capacity, pore size, microstructure, morphology, biodegradation kinetics, rheological properties, and mechanical properties. This multidimensional approach provides clinicians and researchers with a comprehensive biomaterial assessment necessary for scaffold design and selection.

Second, the review captures substantial compositional diversity across hydrogel formulations, ranging from pure gelatine-based systems to complex composite hydrogels incorporating bioceramics, nanofibres, and bioactive molecules. This breadth enables the identification of composition–property relationships that guide material optimisation. For example, incorporating β-TCP as a reinforcement increased compressive strength linearly from 0.4 ± 0.14 MPa in pure gelatine scaffolds to 11.45 ± 1.96 MPa at 60% β-TCP, providing clear, evidence-based guidance for material selection.

Third, the review establishes consistent methodological frameworks for assessing multiple physical properties, enabling quantitative comparison across studies. The detailed documentation of pore size ranges (4–800 micrometres), swelling ratios (6–8 times), and degradation profiles across rapid, moderate, and slow categories enables the systematic evaluation of how material composition affects physical and mechanical characteristics.

Despite these strengths, several limitations warrant acknowledgement. First, the review is constrained by the heterogeneity of measurement methodologies across the included studies. Biodegradation studies, for instance, employed varying conditions. Some utilised simulated body fluid, while others used enzymatic solutions, making direct comparative analysis challenging. Similarly, rheological testing employed differing shear-rate ranges (from 0.1 to 100 s^−1^ to 10^−2^ to 10^4^ s^−1^), complicating standardised interpretation. Therefore, the establishment of universally accepted and standardised testing protocols is strongly recommended to improve reproducibility, facilitate meaningful cross-study comparisons, and accelerate regulatory approval and clinical translation of 3D-bioprinted hydrogel scaffolds for dental applications.

Second, the review lacks comprehensive in vivo validation data. Although the included studies extensively characterise physical and mechanical properties in vitro, the translation of these properties into clinical tissue regeneration outcomes remains underexplored. This gap limits clinical prognostication about which specific mechanical properties optimally support periodontal, bone, or dental pulp regeneration in real-world clinical contexts. Furthermore, most currently available in vivo studies involve relatively short observation periods and may not adequately reflect the complex biological and biomechanical environment of the oral cavity. Consequently, the long-term durability, biodegradation behaviour, vascularisation potential, tissue integration, and host immune responses associated with these scaffolds remain insufficiently understood. Future studies should therefore prioritise extended long-term in vivo investigations and well-designed clinical trials to determine whether favourable in vitro physical, mechanical, and rheological properties can consistently translate into predictable and stable regenerative outcomes over time.

Third, the review is constrained by inconsistent reporting of long-term stability assessments. Most in vitro biodegradation studies terminate at 28 days or fewer, whereas many tissue engineering applications require sustained structural support for longer periods. The limited data on extended degradation timescales (>16 weeks) further restrict understanding of scaffold performance in chronic tissue regeneration scenarios. Long-term in vivo validation studies are therefore essential to evaluate scaffold degradation kinetics, mechanical stability, and regenerative predictability under clinically relevant conditions.

Fourth, the review does not comprehensively address concerns about scalability and manufacturing consistency. While laboratory-scale 3D-bioprinting optimisation is well documented, the review lacks an extensive discussion of industrial-scale fabrication challenges, cost considerations, and the regulatory pathways required for clinical translation. Additionally, the absence of standardised manufacturing and quality control protocols may further hinder reproducibility and commercial scalability. Future efforts should focus on developing automated, cost-effective, and standardised manufacturing workflows capable of producing reproducible scaffolds with consistent biological and mechanical performance.

## 6. Future Directions

Advancing the field of 3D-bioprinted hydrogel scaffolds for dental applications requires several strategic research directions to enable successful clinical translation and commercial adoption. First, standardisation and harmonisation of bioink formulations, printing parameters, and characterisation protocols should be prioritised to enable reliable comparisons between studies and improve reproducibility across research settings. Establishing consensus on conditions for measuring biodegradation, enzymatic degradation protocols, pH buffer systems, and mechanical testing methodologies would enable meaningful cross-study comparisons and improve the reliability of current findings. Similarly, standardising rheological assessment protocols would clarify the relationship between printability parameters and final scaffold performance.

Next, prospective research should focus on developing predictive in vitro characterisation systems that more accurately replicate the dynamic oral environment. Current studies largely rely on simplified laboratory conditions that may not adequately reflect the complex biomechanical loading, salivary contamination, microbial biofilms, and host immune responses encountered in clinical settings. Future studies should therefore incorporate advanced biological models, including co-culture systems, immune-responsive models, and patient-derived cells, to better simulate tissue-specific responses. The integration of computational modelling, finite element analysis, artificial intelligence (AI), machine learning, and digital simulation technologies may further optimise scaffold architecture, predict mechanical behaviour, analyse stress distribution, and reduce trial-and-error during scaffold development. In particular, computational modelling may enable prediction of degradation kinetics, nutrient diffusion, vascularisation potential, and tissue–scaffold interactions prior to in vivo implementation, thereby accelerating scaffold optimisation and reducing experimental costs.

In addition, greater emphasis should be placed on long-term in vivo validation studies beyond 12–16 weeks, as current evidence remains limited regarding the durability, biodegradation behaviour, vascularisation potential, and regenerative stability of 3D-bioprinted hydrogels in the oral cavity. Extended in vivo and clinical studies are essential to determine whether favourable in vitro mechanical and rheological properties consistently translate into predictable outcomes in periodontal, alveolar bone, and dental pulp regeneration. These studies should also evaluate clinically relevant endpoints, including tissue integration, scaffold degradation kinetics, inflammatory responses, patient-reported outcomes, and functional tissue restoration.

Another significant future trajectory is to address regulatory and translational challenges for 3D-bioprinted dental constructs. As many bioprinted scaffolds integrate biomaterials, living cells, growth factors, and bioactive molecules, they may be classified as combination products or advanced therapy medicinal products, which require rigorous regulatory scrutiny. Consequently, future research should focus on establishing universally accepted standards for bioink characterisation, sterility assurance, print fidelity, biocompatibility, degradation behaviour, and manufacturing reproducibility to facilitate approval by regulatory agencies such as the Food and Drug Administration and the European Medicines Agency. Collaboration among researchers, clinicians, engineers, regulatory authorities, and industry stakeholders will be indispensable for developing clinically applicable frameworks to ensure safe and reproducible implementation.

In parallel, personalised bioprinting approaches are expected to be a major future direction in regenerative dentistry. The integration of patient-specific imaging modalities, such as cone beam computed tomography (CBCT), intraoral scanning, and computer-aided design/computer-aided manufacturing (CAD/CAM) workflows, may enable the fabrication of customised hydrogel scaffolds tailored to individual defect morphology and biomechanical requirements. Such personalised constructs may improve scaffold adaptation, tissue integration, and the predictability of regeneration, particularly for complex periodontal, alveolar bone, and craniofacial defects. Furthermore, combining personalised bioprinting with AI-driven design optimisation may facilitate the development of precision regenerative therapies that account for patient-specific anatomical, biological, and functional variations.

Manufacturing scalability and commercial feasibility remain critical barriers to widespread clinical adoption. Most studies are conducted under laboratory-scale conditions using customised fabrication protocols that may not translate easily to large-scale production. Future work should focus on developing automated, standardised, and cost-effective bioprinting workflows that produce reproducible scaffolds while maintaining sterility, cell viability, and mechanical consistency. Optimising chairside bioprinting technologies and patient-specific digital workflows may further enhance the practicality of integrating 3D bioprinting into routine dental practice.

Ultimately, the successful translation and commercialisation of 3D-bioprinted hydrogels for dental applications will depend on demonstrating long-term clinical predictability, cost-effectiveness, regulatory compliance, and clear advantages over existing regenerative therapies. Continued interdisciplinary collaboration and the integration of emerging digital technologies are expected to play a pivotal role in advancing the next generation of personalised regenerative dental therapies.

## 7. Conclusions

This scoping review comprehensively synthesises physical and mechanical characterisation data on 3D-bioprinted hydrogel scaffolds for dental tissue engineering, establishing that material composition directly and predictably influences mechanical properties, degradation kinetics, and structural architecture. The evidence collectively demonstrates that strategic material selection, such as incorporating bioceramics, nanofibres, or polymer blending, enables systematic modulation of scaffold properties to match specific tissue requirements.

Specifically, gelatine-based formulations reinforced with β-TCP achieve compressive strengths within the cancellous bone range, while composite GelMA/PEGDA systems demonstrate tunable stiffness, enabling application-specific customisation. Biodegradation profiles can be engineered to range from rapid (complete degradation by day 7) to slow (sustained structural integrity >16 weeks), permitting temporal optimisation for specific tissue regeneration timescales.

However, significant gaps remain between extensive biological assessments of physical and mechanical characterisation and clinical implementation. Translating measured properties into predictable tissue regeneration outcomes requires systematic in vivo validation studies that explicitly correlate mechanical parameters with functional tissue formation. Standardisation of measurement methodologies, extended temporal characterisation studies, and the integration of computational modelling approaches are essential future priorities.

The successful clinical translation of 3D-bioprinted hydrogel scaffolds for dental tissue regeneration depends on this iterative cycle of physical and mechanical optimisation, rigorous in vivo validation, and systematic pathway development towards regulatory approval. This scoping review provides the physical and mechanical foundation for such clinical translation efforts, offering evidence-based guidance on material selection and highlighting critical knowledge gaps that must be resolved before widespread clinical adoption becomes feasible.

## Figures and Tables

**Table 1:** Three-dimensional bioprinting techniques and strategies for dental applications.

Tissue Type	Bioprinting Technique and 3D Bioprinter	Hydrogel Type	Comparison Groups	Crosslinking Types	Assessments	Author
Bone	Extrusion: Custom built, name or brand unspecified	GelMA and polyethylene glycol dimethacrylate (PEGDA)	Saline group (*n* = 10), hydrogel-treated group (*n* = 10), and PDLSC/hydrogel-treated group (*n* = 10)	Photocrosslinking (UV) of GeIMA	Physical: Morphology, swelling ratio, and degradation Mechanical: Compressive moduli	Ma et al., 2017 [66]
Bone	Extrusion: 3D RegenHU, Villaz-St-Pierre, Switzerland	ECM hydrogel and amorphous magnesium phosphate (AMP)	AMP-free counterpart	NA	Rheology: Viscosity Physical: Morphology Printability	Dubey et al., 2020 [75]
Bone	Extrusion: Korea Institute of Machinery and Materials, Korea	Gelatine and β-TCP nanoparticles	Gelatine, Gel80/tricalcium phosphate20, Gel60/tricalcium phostphate40, and Gel40/tricalcium phosphate60 hydrogels	Chemical crosslinking: Crosslinked at room temperature in 25% glutaraldehyde vapor for 24 h	Rheology: Viscosity and storage modulus Physical: Degradation Mechanical: Compressive strength Printability	Jeong et al., 2020 [71]
Bone	Extrusion: Regenovo, Hangzhou, China	Gelatine (Gel), sodium alginate (SA), and 58S bioactive glass (58S BG)	Control group (did not contain any implant material) and group with Gel/SA/58S BG scaffolds	Chemical crosslinking (scaffolds were soaked in 10% CaCl_2_ solution, crosslinked for 10 min, and then further crosslinked in 0.25% glutaraldehyde (GTA) solution for 30 min)	Rheology: Elasticity Physical: Degradation Mechanical: Compressive strength test, Young’s modulus	Tu et al., 2023 [64]
Bone	Extrusion: BioX, Cellink, Gothenburg, Sweden	MXene-incorporated GelMA and hyaluronic acid methacryloyl (HAMA)	GelMA/HAMA bioinks with and without MXene nanoparticles	Photocrosslinking (addition of a photoinitiator, specifically 2-hydroxy-4′-(2-hydroxyethoxy) -2-methylpropiophenone (Irgacure 2959))	Rheology: Elastic and viscous modulus Physical: Morphology Mechanical: Compressive strength	Lee et al., 2023 [72]
Bone	Digital light processing: (nanoArch S240, BMF Material Technology Inc., China)	Glycidyl methacrylate (GMA)-modified epsilon-poly-L-lysine (EPLGMA)	Group 1: Control group (no scaffold + no bacteria). Group 2: No scaffold + *Porphyromonas gingivalis* (P.g) intervention. Group 3: EPLGMA scaffold + P.g intervention. Group 4: EPLGMA@MDSCs scaffold + P.g intervention. Group 5: EPM scaffold + P.g intervention	Photocrosslinking	Rheology: Swelling rate Physical: Microstructure porosity, morphology, and degradation Mechanical: Young’s modulus	Yu et al., 2024 [67]
Bone and Dentine	Extrusion: Unspecified	BMP-GelMA hydrogel and GelMA hydrogels	GelMA and BMP-GelMA hydrogels	UV crosslinked at 200 mV cm^−2^ for 2 min	Rheology: Storage modulus Physical: Swelling and degradation Mechanical: Compressive strength, Young’s modulus Printability	Park et al., 2020 [76]
Bone and Dentine	Extrusion: A three-axis robot system (DTR3-2210 T-SG; DASA-Robot) equipped with a dispensing system (AD-3000C, Ugin-tech)	Collagen type 1 or decellularised extracellular matrix (dECM) derived from porcine bone with β-TCP	dECM-based biocomposites vs. collagen-based biocomposites (CS: pure collagen/hDPSCs, CTS-20: collagen/β-TCP-20 wt %/hDPSCs, CTS-40: collagen/β-TCP-40 wt%/hDPSCs) with various β-TCP concentrations (0, 20, and 40 wt%)	Chemical crosslinking genipin (1 mM genipin solution). This process was carried out in a medium for 30 min at 37 °C with 5% CO_2_)	Rheology: Shear stress, stiffness, storage, and elastic modulus Physical: Pore size Mechanical: Compressive modulus Printability	Kim et al., 2022 [73]
Bone, Dentine, and Pulp	Extrusion: Modified Hyrel 3D, Norcross, GA, USA	Sodium alginate (3% *w*/*v*) hydrogels with dentine matrix (2:1, 1:1, or 1:2 by volume)	3% alginate, 2:1, 1:1, and 1:2 Alg-Dent hydrogel	Ionic crosslinking (with 0.3 M CaCl_2_)	Rheology: Shear rate Mechanical: Compressive modulus Printability	Athirasala et al., 2018 [77]
Bone and Cementum	Extrusion: 3D Bioplotter Envisiontec, Germany	Sodium alginate (SA), gelatine (Gel), and nano-hydroxyapatite (na-HA)	Two hydrogels: One of 3 wt/vol% sodium alginate (SA) as the control group and the other of 3 wt/vol% SA/7 wt/vol% gelatine/5 wt/vol% nano-hydroxyapatite as the experimental group (S3G7H5)	Ionic crosslink: Crosslinked for 5 min with 2 wt/vol% calcium chloride with a concentration of 180.2 mM (the sodium alginate (SA) component of the hydrogel can be physically crosslinked by calcium ions at room temperature (24–25 °C))	Rheology: Shear rate, shear viscosity Physical: Morphology, swelling Mechanical: Compressive stress, Young’s modulus	Tian et al., 2021 [65]
Bone and Periodontal	Extrusion Direct Ink Writing 3D-Bioplotter System, EnvisionTEC, Germany	Hybrid bioink composed of GelMA and dECM	GelMA, GelMA + 2.5dECM, GelMA + 5dECM, and GelMA + 10dECM	Photocrosslinking polymerisation of GelMA	Rheology: Storage modulus, loss modulus, and dynamic modulus Physical: Swelling ratio, degradation rate, morphology Mechanical: Stiffness Printability: Extrusion pressure, shape fidelity	Yang et al., 2023 [70]
Periodontal	Extrusion: BioScaffolder 3.1 GeSiM, Germany	Gelatine methacryloyl (GelMA)	5, 10, 12.5, 15, and 20% (*w*/*v*) GelMA hydrogel	Photocrosslinking of GeIMA (use of photoinitiators such as Irgacure 2959 (IC 2959) and lithium phenyl-2,4,6-trimethylbenzoylphosphinate (LAP) to initiate the crosslinking process upon exposure to UV light)	Rheology: Storage modulus, viscous modulus, viscosity, Physical: Fibre spacing Mechanical: Stiffness Printability	Raveendran et al., 2019 [79]
Periodontal	Extrusion: Unspecified	Chitosan (CS), sodium alginate (AL), and carbon nanotubes (CNTs)	Group A (control group: w/o carbon nanotubes), Group B (0.1% CNT/CS/AL), Group C (0.25% CNT/CS/AL), Group D (0.5% CNT/CS/AL), and Group E (1% CNT/CS/AL)	Chemical crosslinking; 0.5 mol/L of HCL for 12 h, and then soaking in deionised water to remove excess hydrochloric acid	Physical: Swelling ratio, pore size, morphology, degradation Mechanical: Compressive strength	Suo et al., 2023 [69]
Periodontal	Extrusion: Bio-Architect^®^ WS” Regenovo, Hangzhou, China	GelMA, sodium alginate (SA), and bioactive glass microsphere (BGM)	(1) Non-implanted scaffold (blank group), (2) GelMA/SA/BGM scaffold (scaffold group), (3) GelMA/SA/BGM scaffold containing BMP2 and PDGF (BMP2/PDGF group), and (4) cell-laden GelMA/SA/BGM scaffold containing BMP2 and PDGF (mBMSC/BMP2/PDGF group)	Chemical crosslinked with 4%CaCl_2_ for 5 min	Rheology: Elastic modulus Physical: Swelling behaviour, degradation rate Mechanical: Compressive modulus Printability	Miao et al., 2023 [78]
Periodontal, Dentine, and Pulp	Extrusion: Homemade bioprinting system	Demineralised dentine matrix (DDM) and fibrinogen–gelatine mixture	1%, 3%, 5%, and 10% *w*/*v* DDMp concentration	Chemical fibrinogen crosslinking with thrombin solution (10 U/mL)	Rheology: Shear thinning behaviour, viscosity Mechanical: Compressive modulus Printability	Han et al., 2021 [74]
Enamel	Extrusion: 3D Bioplotter EnvisionTEC GmbH, Gladbeck, Germany	Carboxymethyl chitosan (CMC) and alginate (Alg)	Alg2–CMC4 Alg3–CMC3% Alg4–CMC2%	Dual crosslinking mechanism (physical and ionic crosslinking via Ca+ ions, in which ions facilitate the bonding between polymer chains, differing from chemical crosslinking, which involves covalent bonds)	Physical: Swelling behaviour, degradation rate Mechanical: Elastic modulus Printability	Mohabatpour et al., 2022 [83]
Dentine	Extrusion: BioX, Cellink Gothenburg, Sweden	Calcium silicate (CS) in a GelMA hydrogel	CS0, CS5, and CS10 scaffolds: These groups represent scaffolds with 0%, 5%, and 10% calcium silicate, respectively	Photocrosslinking of GelMA	Rheology: Storage modulus and elastic modulus Physical: Degradation rate Mechanical: stress–strain, Young’s modulus, stiffness Printability	Lin et al., 2021 [82]
Pulp	Extrusion: BioX, Cellink, Gothenburg, Sweden	Gelatine and alginate (GA), enriched in either hydroxyapatite (GAHA) or hydroxyapatite and plasma rich in growth factor (GAHAP)	Gelatine/alginate(GA), gelatine/alginate/hydroxyapatite (GAHA), and gelatine/alginate/hydroxyapatite/PRGF(GAHAP)	Ionic crosslinking: Calcium ions (Ca^2+^) interact with the alginate to form a gel network (crosslinked with 100 mM calcium chloride for 30 min at 37 °C)	Rheology: Viscosity Physical: Swelling capacity, porosity, morphology, degradation rate Mechanical: Young’s modulus	Anitua et al., 2022 [84]
Soft tissues in the dentoalveolar region	Extrusion: 3D Discovery bioprinter RegenHU, Switzerland	Maleic anhydride-grafted chitosan (MA-C) + gelatine	3%(*w*/*v*) gelatine and 1.5%(*w*/*v*) chitosan–MA with 5 different genipin concentrations (gel control, 5 genipin concentrations: 0.5%, 1.0%, 1.5%, 2.0%, and 2.5%)	Chemical crosslinking (using genipin, a natural crosslinking agent)	Physical: Swelling ratio, pore diameter, porosity, morphology, and degradation rate Rheology: Storage and loss modulus, viscosity, Printing fidelity	Amoli et al., 2022 [81]
Dental tissues	Microextrusion, Custom-built air pressure-driven printer, Inkjet	GeIMA with dextran (to form aqueous two phase system @ ATPS)	ICP, NOP, and RDP hydrogels	UV photocrosslinking	Physical: Swelling capacity, pore size, porosity, morphology, degradation rate Rheology: Shear rate, elastic modulus, viscous modulus Mechanical: Compressive strength, Young’s modulus, stiffness Printability	Messaoud et al., 2023 [80]
Dental tissues	Extrusion: Bio-Architect, Regenovo Biotechnology Co. Ltd., Hangzhou, China	Cellulose nanofibrils (CNFs) and gelatine (GEL)	GEL-5, 3–CNF/GEL-5, 5–CNF/GEL-5, 10–CNF/GEL-5, 15– CNF/GEL-5, and 20–CNF/GEL-5, 10–CNF/GEL-3 and 10–CNF/GEL-7	Chemical crosslinking: genipin	Rheology: Shear rate, viscosity, storage modulus Physical: Pore size, porosity, morphology Mechanical: Compressive breaking strength, tensile resilience	Jiang et al., 2018 [68]

24 h, twenty-four hours; 58S BG, 58S bioactive glass; Alg, alginate; Alg-Dent, alginate–dentine; AMP, amorphous magnesium phosphate; AL, sodium alginate; ATPS, aqueous two-phase system; β-TCP, beta-tricalcium phosphate; BGM, bioactive glass microsphere; BMP2, bone morphogenic protein 2; BMP, bone morphogenic protein; Ca+, calcium ions; CaCl_2_, calcium chloride; CS, calcium silicate; CNTs, carbon nanotubes; CS, chitosan; CMC, carboxymethyl chitosan; CNFs, cellulose nanofibrils; DDM, demineralised dentine matrix; dECM, decellularised extracellular matrix; ECM, extracellular matrix; EPLGMA, glycidyl methacrylate (GMA)-modified epsilon-poly-L-lysine; GA, gelatine and alginate; GAHA, gelatine and alginate enriched in hydroxyapatite; GAHAP, gelatine, alginate, hydroxyapatite, and plasma rich in growth factor; GEL, gelatine; Gel, gelatine; GelMA, gelatine methacrylate; Gel80/tricalcium phosphate20, 80% gelatine and 20% beta-tricalcium phosphate; Gel60/tricalcium phosphate40, 60% gelatine and 40% beta-tricalcium phosphate; Gel40/tricalcium phosphate60 hydrogels, 40% gelatine and 60% beta-tricalcium phosphate; GTA, glutaraldehyde; HAMA, hyaluronic acid methacryloyl; ICP, interconnected porous; LAP, lithium phenyl-2,4,6-trimethylbenzoylphosphinate; MA-C, maleic anhydride-grafted chitosan; mBMSCs, mouse bone marrow mesenchymal stem cells; NA, not available; na-HA, nano-hydroxyapatite; NOP, non-porous; PEGDA, polyethylene glycol dimethacrylate; PDGF, platelet-derived growth factor; P.g, *Porphyromonas gingivalis*; RDP, regular-disconnected porous; SA, sodium alginate; and *w*/*v*, weight/volume.

**Table 2:** Three-dimensional-printed hydrogel printing parameters.

Author	Bioprinting Technique and 3D Bioprinter	Gelation Time and Temperature	Printing Parameters	Dimensions of Scaffold
Ma et al., 2017 [66]	Extrusion: Custom built, name or brand unspecified	NA	Nozzle Size: 150 μm Pressure: 50 kPa	NA
Athirasala et al., 2018 [77]	Extrusion: Modified Hyrel 3D, Norcross, GA, USA	NA	Nozzle Size: A coaxial nozzle with a 26G inner needle and a 19G outer needle Printer feed rate: 0.5–0.8 units Flow rate: 45 µL/min Crosslinking: A CaCl_2_ solution was simultaneously pumped into the outer nozzle using a syringe pump (NE300, New Era Pump Systems Inc). Contact between the bioink and the calcium chloride streams at the tip of the nozzle resulted in covalent crosslinking of the bioink and the formation of a fibre	5 × 2 mm discs
Jiang et al., 2018 [68]	Extrusion: Bio-Architect, Regenovo Biotechnology Co. Ltd., from Hangzhou, China	Gelation time: 40 °C for 1 h Gelation temperature: Lower than the T_sol-gel:_ Gel-5: 23.6 °C 10–CNF/GEL-3: 26.4 °C 10–CNF/GEL-5: 30.1 °C 10–CNF/GEL-7: 32.6 °C	Nozzle size: 210 μm Printing pressure: 0.28 MPa Printing speed: 10 mm.s^−1^ Barrel temperatures: 15 °C (best for the pores to remain relatively stable) Work stage temperatures: 10 °C (gives off the highest final struts height, i.e., 15.0 mm) Viscosity: ○ 10–CNF/GEL-3: 0–30 Pa.s ○ 10–CNF/GEL5: 0–65 Pa.s ○ 10–CNF/GEL-7: 0–100 Pa.s (Ideal: 4–30 Pa.s)	NA
Raveendran et al., 2019 [79]	Extrusion: BioScaffolder 3.1 from GeSiM, Germany	Gelation temperature: 37 °C water bath Gelation time: 10 min cooling for thermal	Nozzle Size: 25G needle (optimal for maintaining high cell viability) GelMA concentration (printable at room temperature): ○ Using 25G dispensing tip: only 12.5% and 15% ○ Using 27G dispensing tip: only 10%, 12.5%, and 15% Crosslinking temperature: Cooled to room temperature (~25 °C) Crosslinking time: 20 min to allow thermal crosslinking before being transferred to printing cartridge	5 mm × 5 mm with 4 layers and 0.8 mm fibre spacing
Dubey et al., 2020 [75]	Extrusion: 3D RegenHU, Villaz-St-Pierre, Switzerland	NA	Extrusion pressure: ○ ECM: 15 mm/s ○ ECM/0.5AMP: 16 mm/s ○ ECM/1.0AMP: 20 mm/s Dosing distance (distance for material deposition): ○ ECM: 1.1 mm ○ ECM/0.5AMP: 0.1 mm ○ ECM/1.0AMP: 0.16 mm ○ Valve opening time ○ ECM: 110 µs ○ ECM/0.5AMP: 110 µs ○ ECM/1.0MP: 110 µs The bioink ECM/1.0AMP (lower viscosity) composition required a higher feed rate and lower extrusion pressure for desirable print outcomes when compared to the ECM/0.5AMP (due to the low storage modulus of the ECM/1.0AMP bioink)	NA
Jeong et al., 2020 [71]	Extrusion: Korea Institute of Machinery and Materials, Korea	Gelation temperature: 31 °C	Pressure (uniform extrusion): ○ Gelatine: 230 kPa ○ G80T20: 360 kPa ○ G60T40: 380 kPa ○ G40T60: 430 kPa Stiffness: Highest with G40T60 (gelatine/tricalcium phosphate: 40/60) Temperature: <31 °C (at room temperature) Nozzle size: 21G (produces strand thickness of 500 µm)	7 mm × 7 mm × 3 mm
Park et al., 2020 [76]	Extrusion: unspecified	NA	Extrusion pressure: 130 to 160 kPa Printing temperature: 22 °C Cooling temperature: 18 °C Nozzle Size (inner diameter): 330 µm	5 × 5 × 2 mm^3^
Han et al., 2021 [74]	Extrusion: Homemade bioprinting system	Gelation temperature: 4 °C Gelation time: 10 min	Nozzle diameter: 300 µm Dispensing rate: DDMp bioink: 0.5735 µL/s Printing speed: DDMp bioink: 50 mm/min Environmental condition: Temperature of enclosure maintained at 18 °C Dispensing pressure (with PCL): 200 KPa Printing temperature (with PCL): 85 °C Post-printing treatment: After printing, the DDMp bioink was crosslinked using thrombin solution (10 U/mL) at room temperature for 30–45 min	5 × 5 × 5 mm^3^
Lin et al., 2021 [82]	Extrusion: BioX, Cellink from Gothenburg, Sweden	Gelation temperature: CS0: 25.7 °C CS5: 25.4 °C, CS10: 25.2 °C Gelation time: 90 s	Nozzle size: 30 G Printing pressure: approximately 180 kPa Printing rate: 20 mm/s Crosslinking: Using UV 405 nm light curing for a duration of 90 s	NA
Tian et al., 2021 [65]	Extrusion: 3D Bioplotter from Envisiontec, Germany	Gelation temperature: 24–25 °C Gelation time: 5 min	Needle diameter: 400 μm Printing speed: 6 mm/s Dispensing pressure: 0.2 MPa Fibre spacing: 5 mm Crosslinking: After printing, the cell-free biocomposite scaffold was crosslinked for 5 min with 2 wt/vol% CaCl_2_ solution, which had a concentration of 180.2 mM. The bioscaffolds were then washed twice with PBS, freeze-dried, and sealed for storage	NA
Amoli et al., 2022 [81]	Extrusion: RegenHU, Switzerland	NA (above room temperature)	Nozzle size: 410 µm (22 G) Printing temperature: 20 °C	NA
Anitua et al., 2022 [84]	Extrusion: BioX, Cellink, Gothenburg, Sweden	NA	Needle diameter: ○ GA: 0.20 mm ○ GAHA and GAHAP: 0.25 mm Extrusion speed: 4 mm/s Temperature: ○ Injection head for GA: 45 °C ○ Injection head for GAHA and GAHAP: 100 °C ○ Printing bed: 4 °C Post-printing processing crosslinking: After extrusion, the alginate in the structures was crosslinked using 100 mM calcium chloride for 30 min at 37 °C Freezing and lyophilization: The matrices were then frozen at −80 °C and subsequently lyophilised	NA
Kim et al., 2022 [73]	Extrusion: A three-axis robot system (DTR3-2210 T-SG; DASA-Robot) equipped with a dispensing system (AD-3000C, Ugin-tech)	Gelation temperature: 38 °C	Needle size: 25G Nozzle size (inner diameter): 250 µm Nozzle moving speed: 10 mm/s Barrel temperature: 10 °C Working plate temperature: 38 °C Pneumatic pressure: ○ CS: 14 kPa ○ CTS-10: 15 kPa ○ CTS-20: 17 kPa (good cell viability and stable 3D structural integrity) ○ CTS-30: 24 kPa ○ CTS-40: 26 kPa	10 × 10 × 1.3 mm^3^
Mohabatpour et al., 2022 [83]	Extrusion: 3D Bioplotter EnvisionTEC GmbH, Gladbeck, Germany	NA	Nozzle Size: 27 G, 200 µm (inner diameter) Printing speed: 20 mm/s Pressure (higher alginate concentration, higher viscosity, greater extrusion pressure): ○ Alg2–CMC4%: 0.2 bar ○ Alg3–CMC3%: 0.4 bar ○ Alg4–CMC2%: 0.7 bar Crosslinking: Printing was performed directly into a crosslinker bath containing 50 mM CaCl_2_ solution with 0.1% (*w*/*v*) polyethyleneimine (PEI). After printing, the crosslinking solution was replaced with fresh 100 mM CaCl_2_ for 10 min to further crosslink the scaffolds	10 mm × 10 mm × 5 mm, with 31 layers
Lee et al., 2023 [72]	Extrusion: BioX, Cellink, Gothenburg, Sweden	Gelation temperature: water bath at 37 °C Gelation time: 6 h	Nozzle size (thickness): 580 μm Pressure: 20 kPa Temperature (print bed and nozzle): 15 °C Nozzle to bed distance: 100 µm Flow Rate: 6 mm/s Infill density: 40%	2 cm × 2 cm, 4 layers
Miao et al., 2023 [78]	Extrusion: Bio-Architect^®^ WS, Regenovo, Hangzhou, China	NA	Nozzle diameter: 400 µm Grid distance: 1 mm Temperature: ○ Ink: 20 °C ○ Printing platform: 5 °C Compression rate: 0.5 mm Crosslinking: The scaffolds were crosslinked in a 4% CaCl_2_ solution for 5 min and then irradiated under 365 nm UV light for 30 s to further solidify the structure	Dimensions were: Cell viability assay: 10 mm × 10 mm with two layers Cell proliferation assessments: 10 mm × 10 mm with four layers
Messaoud et al., 2023 [80]	Microextrusion, custom-built air pressure-driven printer, inkjet	Gelation temperature: 37 °C Gelation time: Within seconds	Custom built air-pressure printer: Printing pressure: 0.05 bar Needle size: Inner diameter of 0.5 mm and 0.8 mm Extrusion pressure: 0.1, 0.2, and 0.4 bar Inkjet printer: Diameter: 450 μm Opening times: 450 μs Printing temperature: 37 °C Microextrusion printer: Printing pressure: 0.8 bar Needle size: 25 G Printing temperature: 4 °C Photocured: Using UV LED lamp (30 Watts, 365 nm)	Shape: Cylindrical form Diameter: 6 mm Height: 6 mm
Suo et al., 2023 [69]	Extrusion: Unspecified	NA	Nozzle size/printing diameter: 260 μm Bottom plate forming temperature: −20 °C Ordinary feed speed: 10 mm/s Ordinary extrusion speed: 2 mm/s First layer printing height correction: 0.7 mm Printing layer thickness: 0.32 mm Number of printing layers: 7	After crosslinking: (30 mm × 30 mm × 2 mm) Preparation for cell culture and biocompatibility tests: (0.5 cm × 0.5 cm × 2 mm) Preparation for compressive strength test: (1 cm × 1 cm × 2 mm)
Tu et al., 2023 [64]	Extrusion: Regenovo, Hangzhou, China	NA	Needle diameter: 0.4 mm Extrusion pressure: 0.38 MPa Printing speed: 15 mm/s Extrusion temperature: 28 °C Print table temperature: 10 °C to stabilise Nozzle size: 0.4 mm Ratio of gel to SA: 10:4	Average height: 4.5 mm; 10 layers
Yang et al., 2023 [70]	Extrusion: Direct Ink Writing 3D-Bioplotter System from EnvisionTEC, Germany)	Gelation time: May vary depending on the specific formulation and conditions; the use of UV light for a short duration, such as 30 s, was suggested Gelation temperature: NA	PDL module printing parameters (using a Digital Light Projection bioprinter): Module thickness: 400 µm Internal unit dimensions: Internal diameter: 250 µm Width of hydrogel pillar: 150 µm with a 50 µm gap around it Square grid width: 40 µm DLP system settings: The thickness of the module is controlled by sandwiching two narrow silicon membranes between two holders to obtain a gap Alveolar bone module printing parameters: Nozzle size: Inner diameter of 260 μm Needle offset: Along the z-direction: 210 μm Printing speed: 12 mm s^−1^ Printing pressure: ○ GelMA bioink: 90 kPa for the GelMA bioink ○ GelMA + 5dECM bioink: 150 kPa Temperature: ○ Barrel containing the bioink: 27 °C ○ Room temperature: 23 °C UV exposure: 405 nm UV light for 30 s	PDL module: The internal diameter of each unit in the PDL module was 250 μm, which included a hydrogel pillar that was 150 μm wide and a 50 μm gap around it. The square grid used in the PDL module was 40 μm in width Alveolar bone module: The cube structure of the alveolar bone module measured 10 mm × 10 mm × 2.5 mm. This design included an inner strand distance of 0.9 mm and inner strand angles of 0° and 90° to replicate the natural architecture of alveolar bone
Yu et al., 2024 [67]	Digital light processing: (nanoArch S240, BMF Material Technology Inc. in China)	Gelation time: <40 s when exposed to 405 nm UV light Gelation temperature: 37 °C	Environmental conditions: Temperature: 37 °C Pressure: Standard atmospheric pressure (101.325 kPa) Exposure intensity: Blue light (405 nm laser) at 18 mW cm^−2^ with an exposure time of 30 s per layer	4 mm × 4 mm × 1 mm Thickness of each layer: 400 µm No of layers printed: 4 Total thickness: 1600 µm

AMP, amorphous magnesium phosphate; CaCl_2_, calcium chloride; CNFs, cellulose nanofibrils; DDMps, demineralised dentine matrix particles; DLP, digital light processing; ECM, extracellular matrix; G, gauge; G80T20, 80% gelatine and 20% beta-tricalcium phosphate; G60T40, 60% gelatine and 40% beta-tricalcium phosphate; G40T60, 40% gelatine and 60% beta-tricalcium phosphate; GA, gelatine and alginate; GAHA, gelatine and alginate enriched in hydroxyapatite; GAHAP, gelatine, alginate, hydroxyapatite, and plasma rich in growth factor; GEL, gelatine; GelMA, gelatine methacrylate; NA, not available; PBS, phosphate-buffered saline; PCL, polycaprolactone; PDL, periodontal ligament; PEI, polyethyleneimine; T_sol-gel_, temperature at which hydrogel transitions from a sol (liquid-like) state to a gel (solid-like) state; UV, ultraviolet; and *w*/*v*, weight/volume.

**Table 3:** Physical assessments of 3D-printed hydrogels.

Tissue Type	Swelling Ratio/Capacity	Pore Size	Microstructure Porosity	Morphology	In Vitro (Enzymatic) Biodegradation	Author
Bone	As the volume ratio of GelMA-to-PEGDA changes from 1/4 to 4/1, the swelling ratio increases from approximately 750% to 950%. 5/0 has the highest SR: 1200%; 1/4 has the lowest SR: 750%. The SR of GelMA/PEGDA hydrogels varies significantly with the composition	The pore sizes of the GelMA/PEGDA hydrogels vary depending on the volume ratio of GelMA to PEGDA. The pore sizes are as follows: For a 1/4 GelMA/PEGDA ratio, the pore size is approximately 55.7 ± 10.1 μm. For a 2/3 ratio, the pore size is about 96.1 ± 15.5 μm. For a 3/2 ratio, the pore size is around 111.3 ± 20.8 μm. For a 4/1 ratio, the pore size is approximately 160.0 ± 26.4 μm. For a 5/0 ratio (pure GelMA), the pore size is about 248.9 ± 35.5 μm	NA	An inverse correlation between the GelMA-to-PEGDA ratio and the pore size of the hydrogel. As the volume ratio of GelMA decreases and PEGDA increases, the pore size of the hydrogels also decreases	Hydrogels with a higher GelMA content degrade faster. For instance, the 5/0 GelMA hydrogel showed almost complete degradation by 7 days, which was considered too fast for effective tissue regeneration. Hydrogels with GelMA/PEGDA ratios ranging from 4/1 to 1/4 showed significantly slower degradation rates, with mass remaining even after 14 days of incubation. As more PEGDA was incorporated into the hydrogel, and the volume ratio of GelMA-to-PEGDA decreased from 4/1 to 1/4, the degradation rate decreased (*p* < 0.05)	Ma et al., 2017 [66]
Bone	NA	NA	NA	AMP powder consists of micron-sized particles with plate-like structures, contributing to the overall morphology of the bioink ECM had a tight, dense, and wrinkled surface AMP-modified bioink (ECM/AMP) appeared rougher due to the presence of AMP	After 4 weeks of implantation, no remaining AMP particles were observed, indicating a faster degradation of AMP	Dubey et al., 2020 [75]
Bone	NA	NA	Interconnected porous microstructure, uniform distribution of ß-TCP nanoparticles, and controlled architectural features, which include a thickness of 500 µm and a spacing of 700 µm between strands	β-TCP nanoparticles created a rough surface for the scaffold, providing a suitable environment for cell attachment and filopodia production was active on this surface	On the 28th day: Gelatine-only: 16% (highest) G40T60: 1% G60T40: 2% G80T20: 4% Addition of β-TCP reduced the degradation rate	Jeong et al., 2020 [71]
Bone	SR: 0.2 Fast speed of water absorption during the first hour and then had a stable swelling rate of approximately 4.58 after 2 h	~600 μm	Porosity higher than 50%	Porous structure with the presence of pits and multi-level pore structures on the surfaces of the fibres The 58S BG particles were evenly distributed in the scaffold	Degradation rate: 17% (after incubation in SBF for 16 weeks) Structural integrity: Maintained their structure for at least 4 weeks and lost about 15% of their weight after 16 weeks	Tu et al., 2023 [64]
Bone	NA	GelMA: 12.01 ± 1.53 μm GelMA/HAMA: 17.59 ± 3.45 μm GelMA-MXene: 18.73 ± 3.01 μm GelMA/HAMA-MXene: 19.22 ± 3.06 μm	NA	Images revealed that all bioinks, including GelMA, GelMA/HAMA, GelMA-MXene, and GelMA/HAMA-MXene possess microporous structures	NA	Lee et al., 2023 [72]
Bone	ESR of the lyophilised bioink was approximately 7.3%	NA	Net-like porous structures	NA	After 15 days of incubation in PBS, EPLGMA/PDLSC/MDSC-MV (EPM) bioink exhibited a degradation ratio of approximately 20%. This suggests that the bioink has a controlled degradation profile, which is beneficial for its application in tissue engineering	Yu et al., 2024 [67]
Bone and Dentine	SR: GelMA: 12 BMP-GelMA: 12.5	NA	NA	NA	Degradation of the GelMA and BMP-GelMA hydrogel constructs was evaluated by measuring mass loss over time. Both types of constructs showed similar mass loss profiles, i.e., approximately 60% mass loss on day 28	Park et al., 2020 [76]
Bone and Dentine	NA	500 μm	NA	NA	NA	Kim et al., 2022 [73]
Bone, Dentine, and Pulp	NA	NA	NA	NA	NA	Athirasala et al., 2019 [77]
Bone and Cementum	For the S3 and S3G7H5 bioscaffolds, the ESRs were 415.7% and 125.9%	S3 bioscaffold has a larger pore diameter compared to the S3G7H5 bioscaffold	S3 bioscaffold exhibited a larger porosity compared to the S3G7H5 bioscaffold	Colour: S3: Transparent S3G7H5: White Surface: S3: Smooth and flat S3G7H5: Rough (caused by inorganic particles on bioscaffold)	NA	Tian et al., 2021 [65]
Bone and Periodontal	SR: GelMA: 6.2 GelMA + 2.5dECM: 7.5 GelMA + 5dECM: 7 GelMA + 10dECM: 7 The SR of the GelMA hydrogels did not significantly change with the addition of dECM	NA	The study utilised a two-layer scaffold with a specific internal structure, where the distance between adjacent filaments was set at 1.2 mm	Precise lattice-like arrangement of hydrogel pillars and supporting grids, which not only provides structural stability but also guides the directional alignment of encapsulated cells, mimicking natural periodontal fibres	After placing hydrogel samples in a 24-well plate with 0.5 mg mL^−1^ collagenase I at 37 °C, the remaining mass at 24 h was as follows: GelMA: 65% GelMA + 2.5dECM: 20% GelMA + 5dECM: 19% GelMA + 10dECM: 15% Addition of dECM to the GelMA bioink significantly increased the degradation rate. Specifically, the GelMA/dECM bioink degraded more rapidly than the pure GelMA hydrogel, likely due to increased organic content	Yang et al., 2023 [70]
Periodontal	NA	NA	Fibre spacing: 0.8 mm	NA	NA	Raveendran et al., 2019 [79]
Periodontal	SR showed an upward trend initially and became stable after 6 h. CNTs reduced the swelling capacity of the scaffold At the 8th hour: Control group: 17% 1% CNT/CS/AL group: 15% 0.5% CNT/CS/AL group: 14.5% 0.25% CNT/CS/AL group: 13.5% 0.1% CNT/CS/AL group: 13%	400 μm (suitable for cell growth and function)	Fibres had a dimension of approximately 500 μm and the spacing between them was 300–400 μm	Scaffold was a loose and porous structure	At the 21st day: Control group (highest rate): 35.69 ± 2.46% 0.1% CNT/CS/AL group: 34.00 ± 3.01% 0.25% CNT/CS/AL group: 33.01 ± 2.54% 0.5% CNT/CS/AL group: 29.27 ± 2.19% 1% CNT/CS/AL group: 21.15 ± 2.89%	Suo et al., 2022 [69]
Periodontal	Hydration degree values were: GelMA/SA: 85%; GelMA/SA/BGM: 80%	NA	The GelMA/SA scaffold features a relatively intact filament structure, while the GelMA/SA/BGM scaffold contains a large number of small pores within its filaments	Initial microstructure and surface morphology: Connected pore structure: Both lyophilised GelMA/SA and GelMA/SA/BGM scaffolds exhibited a connected pore structure along the depositional direction, along with a similar pore size Filament integrity: The GelMA/SA scaffold showed a relatively intact filament structure. In contrast, the GelMA/SA/BGM scaffold displayed a large number of small pores within its filaments Surface characteristics: At high magnification, the GelMA/SA scaffold had a relatively smooth surface, while the GelMA/SA/BGM scaffold presented a rough surface due to the uniform encapsulation of bioactive glass microsphere (BGM) particles within its matrix Day 7: GelMA/SA: A thin layer of needle-like apatite GelMA/SA/BGM: A thick layer of apatite	Degradation of the GelMA/SA scaffold was faster than the GelMA/SA/BGM scaffold. After 48 h, the mass remaining in the GelMA/SA scaffold was 21.42%, while it was 37.12% for the GelMA/SA/BGM scaffold (due to reduced water absorption capacity of the latter, which limits the rapid adsorption of the collagenase solution and subsequent degradation). These findings suggest that the incorporation of BGM into the scaffold enhances its stability and slows down its degradation rate, which could be beneficial for maintaining scaffold integrity during tissue regeneration	Miao et al., 2023 [78]
Periodontal, Dentine, and Pulp	NA	NA	NA	The prepared DDMps have irregular polygonal shapes in microscale and 2–4 μm diameter dentinal tubules on the surfaces. Average size of the DDMps was approximately 48.13 µm	NA	Han et al., 2021 [74]
Enamel	On day 1, the SR values were: Alg2–CMC4%: 28.12 ± 1.282 Alg3–CMC3%: 23.47 ± 1.249 Alg4–CMC2% 23.50 ± 0.319 By day 7, the SR increased for all groups, with no significant difference between them On day 14, the SR values were: Alg3–CMC3%: 43.16 ± 1.0 (highest) Alg2–CMC4%: 35.69 ± 1.643 (lowest) By day 21, the SR values were: Alg4–CMC2%: 43.58 ± 0.713 (highest)	Pore size: Alg2–CMC4%: 0.716 ± 0.054 mm (significantly larger pore size) Alg3–CMC3%: 0.492 ± 0.037 mm Alg4–CMC2%: 0.453 ± 0.029 mm	The 3D-printed hydrogel scaffolds were observed to have a highly porous structure with well-defined intersections Effect of CMC ratio: Scaffolds with higher ratios of carboxymethyl chitosan (CMC) exhibited smaller filament sizes and maintained pores with better square-shaped morphology and larger sizes during the printing process Multilayered structures: The Alg-CMC hydrogels were capable of being printed as multilayered structures with relatively straight strands and adequate porous structures	NA	Alg2–CMC4% scaffolds remaining weight (significantly decreased, *p* < 0.01): Day 1: 94.1% Day 14: 68.42% Day 21: 69.6% Alg3–CMC3% scaffolds remaining weight: Day 1: 101.8% Day 7: 94.68% Day 14: 95.23% Day 21: 86.97% Alg4–CMC2% scaffolds remaining weight (slowest degradation, *p* > 0.05): Weight remained unchanged over 21 days of the degradation test Shape was also maintained in PBS until day 21 It can be concluded that the higher concentration of CMC lead to a higher degradation rate	Mohabatpour et al., 2022 [83]
Dentine	NA	NA	NA	NA	After 28 days of immersion, the weight loss values were: CS0: 12% CS5: 18% CS10: 24%	Lin et al., 2021 [82]
Pulp	Swelling capacity of the scaffolds was found to be high, ranging from 80% to 120% of their initial weight Scaffolds containing GAHA and GAHAP showed a lower ability to retain water compared to the GA scaffolds, suggesting that the presence of hydroxyapatite affects the swelling behaviour. At 24 h, the SR values were: GA (highest): 12 GAHA: 9 GAHAP: 10	NA	NA	Pore structure: All scaffolds contained pores. However, the lyophilisation process used in their preparation may have led to irregularly arranged pores due to the formation of ice crystals during freezing, which were then removed through sublimation and desorption processes Surface characteristics: GA: homogenous surface GAHA and GAHAP: Displayed minerals on their surfaces, confirming the presence of hydroxyapatite (HA) Additionally, the GAHAP scaffold showed fibrin remains, indicating the presence of PRGF Transversal structure: GAHA and GAHAP scaffolds had a more compact structure with fewer pores compared to the GA scaffold, suggesting differences in porosity and structural density among the scaffold types	After 7 days: GA (highest): 10% GAHA: 8% GAHAP (lowest): 6% The GAHAP gels exhibited the lowest degradation rate among the tested scaffolds. This means they maintained their structure better over time compared to other formulations like the GA scaffolds, which degraded more quickly under simulated physiological conditions	Anitua et al., 2022 [84]
Soft tissues in dentoalveolar region	SR: 6–8 times Highest within 48 h 0.5% genipin: 8.2 1% genipin: 8 1.5% genipin: 7.8 2% genipin: 7.5 2.5% genipin: 6.8	0.5 wt% genipin: 86.3 ± 8.1 μm (larger) 2.5 wt% genipin: 63.6 ± 6.8 μm (smaller) Pore size can be effectively tuned by adjusting the genipin concentration	NA	Both samples appear in SEM images to have a large degree of porosity, confirming the swelling observed due to the high-water content	Maximum degradation within 14 days of immersion in PBS: 0.5% genipin (highest): 41% 1%genipin: 40% 1.5% genipin: 35% 2% genipin: 31% 2.5% genipin: 20% Differences in genipin concentration lead to significant variations in degradation rates after 7 days, except for certain concentration comparisons (0.5 wt% vs. 1 wt% and 1.5 wt% vs. 2 wt%)	Amoli et al., 2022 [81]
Dental tissues	NA	RDP1: 15 µm RDP2: 28 µm RDP3: 70 µm ICP: 4 to 100 µm	Mean porosities: RDP: 12 µm ICP: 50 µm	GelMA hydrogels exhibited a fibrillar feature RDP hydrogels showed individual disconnected pores ICP hydrogels demonstrated a nanofibrous architecture	NA	Messaoud et al., 2023 [80]
Dental tissues	NA	The initial designed pore size in the printing process was 400 μm, which was reduced to 200 μm as the strut height increased. Pore size of 400 μm is produced at a barrel temperature of: 10 °C (initial strut height~4 mm) 15 °C (initial strut height~5 mm) 20 °C (initial strut height~3 mm) 25 °C (initial strut height~2 mm)	NA	Pure gelatine (GEL) hydrogel had a very loose structure with large pores and smooth-aperture walls As the cellulose nanofibre (CNF) content increased, the formation of smaller ice crystals during lyophilization led to decreased pore sizes and increased structural compactness	NA	Jiang et al., 2018 [68]

58S BG, 58S bioactive glass; Alg, alginate; AL, sodium alginate; AMP, amorphous magnesium phosphate; BGM, bioactive glass microsphere; BMP, bone morphogenic protein; β-TCP, beta-tricalcium phosphate; CMC, carboxymethyl chitosan; CNF, cellulose nanofibre; CNTs, carbon nanotubes; CS, calcium silicate; CS0, bioink with 0% calcium silicate powder; CS5, bioink 5% calcium silicate; CS10, bioink with 10% calcium silicate powder; DDMps, demineralised dentine matrix particles; dECM, decellularised extracellular matrix; ECM, extracellular matrix; EPLGMA, glycidyl methacrylate (GMA)-modified epsilon-poly-L-lysine; ESR, equilibrium swelling ratio; G80T20, 80% gelatine and 20% beta-tricalcium phosphate; G60T40, 60% gelatine and 40% beta-tricalcium phosphate; G40T60, 40% gelatine and 60% beta-tricalcium phosphate; GA, gelatine and alginate; GAHA, gelatine and alginate enriched in hydroxyapatite; GAHAP, gelatine, alginate, hydroxyapatite, and plasma rich in growth factor; GEL, gelatine; GelMA, gelatine methacrylate; HAMA, hyaluronic acid methacryloyl; ICP, interconnected porous; MDSC-MVs, myeloid-derived suppressor cell membrane vesicles; NA, not available; ODM, osteogenic differentiation medium; PBS, phosphate-buffered saline; PDLSCs, periodontal ligament stem cells; PEGDA, polyethylene glycol dimethacrylate; RDP, regular-disconnected porous; S3; bioscaffold composed solely of 3% sodium alginate; S3G7H5, bioscaffold made from 3% sodium alginate, 7% gelatine, and 5% nano-hydroxyapatite; SA, sodium alginate; SEM, scanning electron microscope; SR, swelling ratio; and wt%, weight percentage.

**Table 4:** Rheological properties of 3D-bioprinted hydrogels.

Tissue Type	Shear Rate, Shear Strength, Viscosity	Storage (G′) and Loss Modulus (G″)	Author
Bone	NA	NA	Ma et al., 2017 [66]
Dental tissues	The viscosities of 10–CNF/GEL-3, 10–CNF/GEL-5, and 10–CNF/GEL-7 were measured at shear rates ranging from 0.1 to 100 s^−1^ at 10 °C. All samples exhibited shear-thinning behaviour, meaning their viscosity decreased with increasing shear rate, which is beneficial for 3D printing applications	The G′ increases with higher solid content, indicating improved rigidity and elasticity of the hydrogel	Jiang et al., 2018 [68]
Bone, Dentine, and Pulp	The shear rate varied from 0.1 to 100 s^−1^ during rheological measurements. The 3% alginate hydrogel precursor had the highest viscosity, while the Alg-Dent hydrogel blends had significantly lower viscosities. Despite these differences, the shear-thinning properties were not adversely affected by the inclusion of dentine matrix proteins	NA	Athirasala et al., 2019 [77]
Periodontal	At 1 s^−1^, the viscosity values were: 5% (*w*/*v*) GelMA: 9.7 Pa·s 20% (*w*/*v*) GelMA: 200 Pa·s The viscosity of all of the hydrogel precursors decreased gradually with the increase in shear rate, indicating a flow behaviour under high shear, which made the hydrogel precursors suitable for extrusion	All of the hydrogel precursors had higher G′ than G″, suggesting that the gel precursors have more elasticity than viscosity. The rheological behaviour of GelMA indicates that higher concentrations result in increased viscosity and storage modulus, which suggests a more solid-like behaviour and potentially greater mechanical strength. The G′ and G″ values of the GelMA hydrogel precursors increased with the increase in GelMA concentration 20% (*w*/*v*) GelMA hydrogel has the highest storage modulus (G′:7000 Pa) and viscous modulus (G″:300 Pa) At higher shear strain, the G′ values dropped. At the same time, the G″ value increased, indicating that the GelMA was behaving more similarly to a liquid	Raveendran et al., 2019 [79]
Bone	The ECM hydrogel demonstrated shear-thinning behaviour, meaning its viscosity decreased as the shear rate increased. The addition of ECM/0.5AMP increased the complex viscosity, whereas ECM/1.0AMP decreased the viscosity without negatively impacting the shear-thinning properties	The G′ and G″ values primarily remained higher in all groups, thus indicating a viscoelastic gel behaviour with 0.5% AMP showing the highest value. At 1 rad/s, the G′ for the ECM/0.5AMP is 1083 Pa, indicating a strong viscoelastic gel behaviour (can maintain its shape and structure effectively during and after the printing process) compared to 435 and 260 Pa for only the ECM and ECM/1.0AMP, respectively. The ECM/1.0AMP possesses a G′ value close to the ECM hydrogel, indicating that a similar elastic effect may occur during printing	Dubey et al., 2020 [75]
Bone	On measuring the viscosity according to shear rate (0–100 s^−1^), all groups exhibited shear-thinning behaviour, confirming that the hydrogels were extrudable materials and suitable for printing	At a frequency of 10 Hz, the G′ values were: G40T60: 5000 Pa G60T40: 500 Pa G80T20: 50 Pa Gelatine: 0.3 Pa	Jeong et al., 2020 [71]
Bone and Dentine	NA	The G′ before UV crosslinking GelMA and BMP-GelMA bioinks exhibited a ~2 kPa and a sharp increase to ~4 kPa after UV crosslinking. This indicates that the hydrogels become stiffer and more elastic after crosslinking. The GelMA group had a slightly higher storage modulus than the BMP-GelMA group, although the difference was not statistically significant)	Park et al., 2020 [76]
Periodontal, Dentine, and Pulp	Shear rate was measured during rheological property analysis, ranging from 0.1 to 100 s^−1^ (viscosity peaks at 0.5 s^−1^). The viscosity of the DDMp bioink increased with the concentration of DDM particles. The bioink exhibited shear-thinning properties, meaning its viscosity decreased as the shear rate increased. Shear-thinning behaviour: The DDMp bioink exhibits shear-thinning properties, meaning its viscosity decreases with an increase in shear rate. This behaviour is beneficial for 3D printing as it allows the bioink to flow easily during extrusion and maintain its shape once printed. Viscosity and concentration: The viscosity of the DDMp bioink increases with the concentration of DDM particles. Higher concentrations lead to improved viscosity, which enhances the printability and structural integrity of the printed constructs	NA	Han et al., 2021 [74]
Dentine	NA	Associated stress values where G′ and G″ intersect and T_sol-gel_ were: CS0: 3.31 kPa, 25.7 °C CS5: 5.26 kPa, 25.4 °C CS10: 12.99 kPa, 25.2 °C	Lin et al., 2021 [82]
Bone and Cementum	Shear rate varied from 0.001 to 100 s^−1^ at 25 °C. The shear viscosity decreases non-linearly with the increase in shear rate, and the viscosity of the two hydrogels is within 300 Pa·s. When the shear rate was near 0 s^−1^, the maximum viscosities of S3 and S3G7H5 hydrogels were 118.6 and 248.3 Pa·s, respectively. At the same shear rate, the viscosity of the S3G7H5 composite hydrogel samples is larger than that of the S3 hydrogels. The two hydrogels are shear thinning non-Newtonian fluids	Compression stress: 0.671 MPa; compression elastic modulus of S3: 4.13 MPa, S3G7H5: 8.27 MPa	Tian et al., 2021 [65]
Soft tissues in dental alveolar region	The higher magnitude of the storage modulus over the loss modulus suggests that the material exhibits predominantly elastic behaviour, which implies lower viscosity under the tested conditions	Across all tested scenarios, the materials exhibited higher magnitudes of the G′ compared to the G″, indicating a predominantly elastic behaviour over a wide range of angular frequencies	Amoli et al., 2022 [81]
Pulp	Viscosity range of 0–200 kPa	NA	Anitua et al., 2022 [84]
Bone and Dentine	Shear stress (0.1–1000 Pa, temperature: 25 °C, frequency: 1 Hz) and temperature sweeps (10–50 °C, frequency: 1 Hz, strain: 1%).	The bioink with a higher concentration of bioceramic showed a significantly higher G′ at 25 °C, whereas a similar yield stress (τy) was observed (G′ in Pascals for CS: ~150 Pa; CTS-20: 250 Pa; CTS-40: ~500 Pa) For the temperature sweep result, G′ peaks of the bioinks were observed near 38 °C owing to collagen in the bioink. Gelation was the highest in the pure collagen bioink, and as the ceramic concentration increased, the modulus was significantly reduced owing to the thermal conduction of the embedded ceramics in the bioink	Kim et al., 2022 [73]
Enamel	Among the different hydrogel solutions tested, the Alg2–CMC4% group had the lowest viscosity, requiring the lowest extrusion pressure. In contrast, solutions with higher alginate concentrations, such as Alg3–CMC3% and Alg4–CMC2%, exhibited higher viscosities and required higher extrusion pressures	Alg4–CMC2% (57.08 ± 6.162 kPa) exhibited the highest elastic modulus among all three groups with 2.2- and 9-fold increases compared to Alg3–CMC3% (26.02 ± 3.663 kPa) and Alg2–CMC4% (6.320 ± 1.339 kPa), respectively	Mohabatpour et al., 2022 [83]
Periodontal	NA	Elastic modulus: 1% CNT/CS/AL group: 75 kPa (highest) 0.5% CNT/CS/AL group: 50 kPa 0.25% CNT/CS/AL group: 35 kPa 0.1% CNT/CS/AL group: ~28 kPa Control: 15 kPa This range suggests that the scaffolds possess adequate mechanical strength, which is essential for supporting periodontal tissue regeneration. The highest elastic modulus was observed in the 1% CNT/CS/AL group, indicating that increasing CNT concentration enhances the mechanical properties of the scaffold	Suo et al., 2022 [69]
Bone	The G′ and G″ values suggest that the bioinks have suitable viscosity for supporting cell growth and maintaining structural integrity during and after printing	The G′ values of the GelMA/HAMA and GelMA/HAMA-MXene bioinks were 185.68 ± 12.29 Pa and 206.26 ± 11.40 Pa, respectively, and the G″ values were 2.55 ± 0.59 Pa and 2.99 ± 0.72 Pa, respectively. These values suggest that the incorporation of MXene slightly increases the storage modulus, indicating enhanced elastic properties suitable for supporting cell growth	Lee et al., 2023 [72]
Periodontal	NA	Elastic/compressive modulus: GelMA/SA: 310 kPa and GelMA/SA/BGM: 250 kPa GelMA/SA/BGM scaffold showed a decreased elastic modulus compared to the GelMA/SA scaffold, indicating that the addition of BGMs affects the mechanical properties of the scaffold	Miao et al., 2023 [78]
Dental tissues	Shear rates from 10^−2^ to 10^3^ s^−1^ were tested	The G′ was found to be significantly higher than G″, indicating a predominantly elastic behaviour of the hydrogels. Specifically, the G′ values for the different hydrogel types were approximately: ICP hydrogel: 2.5 kPa RDP hydrogel: 4 kPa NOP hydrogel: 4 kPa	Messaoud et al., 2023 [80]
Bone	Shear thinning behaviour: As the shear rate increases, the viscosity of the material decreases, allowing for easier extrusion through the printer nozzle	NA	Tu et al., 2023 [64]
Bone and Periodontal	Shear rate range tested: 0.1 to 100 s^−1^ Shear viscosity: Bioink exhibited shear-thinning behaviour, meaning that its viscosity decreased with increasing shear rate	The G′ was consistently higher than the G″ across the entire frequency range tested The relationship between the G′ and G″ indicates that the bioink behaves more like an elastic solid than a viscous fluid, which is advantageous for applications requiring shape retention Dynamic modulus: The study highlighted that the dynamic modulus of the GelMA/dECM bioink increased with the concentration of dECM, suggesting that higher concentrations enhance the mechanical properties of the bioink, making it more suitable for 3D bioprinting applications	Yang et al., 2023 [70]
Bone	Only mention of shear rate pertains to the dynamic time-sweep rheological analysis, which indicates that the bioink can be light-cured in less than 40 s upon exposure to 405 nm UV light	NA	Yu et al., 2024 [67]

Alg, alginate; Alg-Dent, alginate–dentine matrix; AL, sodium alginate; AMP, amorphous magnesium phosphate; BGMs, bioactive glass microspheres; BMP, bone morphogenic protein; CMC, carboxymethyl chitosan; CNF, cellulose nanofibre; CNTs, carbon nanotubes; CNT/CS/AL, carbon nanotube/chitosan/sodium alginate; CS, pure collagen/hDPSCs; CTS-20, collagen/20% weight percentage beta-tricalcium phosphate weight with human dental pulp stem cells; CTS-40, collagen/40% weight percentage beta-tricalcium phosphate weight with human dental pulp stem cells; CS0, bioink with 0% calcium silicate powder; CS5, bioink with 5% calcium silicate; CS10, bioink with 10% calcium silicate powder; DDMps, demineralised dentine matrix particles; dECM, decellularised extracellular matrix; ECM, extracellular matrix; G′, elastic modulus; G″, viscous modulus; G80T20, 80% gelatine and 20% beta-tricalcium phosphate; G60T40, 60% gelatine and 40% beta-tricalcium phosphate; G40T60, 40% gelatine and 60% beta-tricalcium phosphate; GEL, gelatine; GelMA, gelatine methacrylate; HAMA, hyaluronic acid methacryloyl; ICP, interconnected porous; NA, not available; NOP, non-porous; RDP, regular-disconnected porous; SA, sodium alginate; UV, ultraviolet; S3; bioscaffold composed solely of 3% sodium alginate; S3G7H5, bioscaffold made from 3% sodium alginate, 7% gelatine, and 5% nano-hydroxyapatite; T_sol-gel_, temperate at which hydrogel transitions from a sol (liquid-like) state to a gel (solid-like) state.

**Table 5:** Mechanical properties of 3D-bioprinted hydrogels.

Tissue Type	Compressive Strength	Young’s Modulus	Stiffness (Under Mechanical)	Tensile Resilience	Author
Bone	Compressive modulus of GelMA/PEGDA in the following ratios: 1/4: 24 kPa (highest) 5/0: 5 kPa (lowest)	NA	Stiffness of GelMA/PEGDA hydrogels (i.e., 5/0 hydrogel) was 4.5 ± 2.3 kPa, which was significantly increased to 13.8 ± 1.7 kPa, 17.9 ± 2.3 kPa, 19.2 ± 2.1 kPa, and 23.5 ± 2.6 kPa as the volume ratio of GelMA-to-PEGDA decreased to 4/1, 3/2, 2/3, and 1/4, respectively	NA	Ma et al., 2017 [66]
Bone	NA	NA	The ECM/0.5AMP has the highest stiffness among the tested formulations, which can influence its printability and mechanical properties	NA	Dubey et al., 2020 [75]
Bone	Gelatine only: 0.4 ± 0.14 MPa (lowest) G80T20 (20% β-TCP): 4.12 ± 0.22 MPa G60T40 (40% β-TCP): 8.41 ± 1.41 MPa G40T60 (60% β-TCP): 11.45 ± 1.96 MPa	NA	NA	NA	Jeong et al., 2020 [71]
Bone	The compressive strength test demonstrated that the load was significantly increased when displacement reached 3 mm	Gel/SA/58S BG scaffolds: 265.80 MPa, which reflects the material’s ability to withstand shear forces without deforming significantly	The stiffness of the scaffold is related to its compressive strength and Young’s modulus. The average Young’s modulus of the scaffolds was measured to be 265.80 MPa, which indicates the stiffness of the material under compression, suggesting that the scaffold has sufficient stiffness to support bone tissue regeneration while maintaining a suitable stress condition for bone restoration	The scaffolds could be compressed to at least 60% of their width and then restored to their original form, indicating good tensile resilience	Tu et al., 2023 [64]
Bone	The compressive strength values were: GelMA: 16.75 ± 1.93 kPa GelMA/HAMA: 17.40 ± 3.38 kPa GelMA-MXene: 26.74 ± 0.64 kPa GelMA/HAMA-MXene: 27.43 ± 2.46 kPa	NA	NA	The use of 2D nanomaterials like MXene in hydrogel-based nanocomposites allows for the creation of structures with adjustable mechanical properties, which can include improved tensile resilience	Lee et al., 2023 [72]
Bone	NA	NA	The bioink possesses suitable stiffness characteristics for effective use in bone regeneration applications	NA	Yu et al., 2024 [67]
Bone and Dentine	NA	NA	The incorporation of BMP mimetic peptides into the GelMA hydrogels does not significantly alter the stiffness of the material, as both the storage modulus and Young’s modulus remain similar between the two groups	NA	Park et al., 2020 [76]
Bone and Dentine	NA	NA	Compressive modulus: The compressive modulus of the CTS-20 scaffold, which contains 20 wt% bioceramic, is significantly higher (27.9 ± 2.2 kPa) compared to the CS scaffold (5.6 ± 0.8 kPa). This increase in stiffness is attributed to the embedded bioceramics (β-TCP) in the bioink	NA	Kim et al., 2022 [73]
Bone, Dentine, and Pulp	NA	NA	The stiffness of the hydrogels is discussed in terms of their compressive modulus. Pure alginate had a compressive modulus of approximately 6 kPa, while the addition of dentine reduced this modulus to about 1 to 2 kPa, indicating a decrease in stiffness with the addition of dentine components	NA	Athirasala et al., 2019 [77]
Bone and Cementum	Compressive stress value of 0.671 MPa. At 60% deformation, the compression stress of the S3 bioscaffold was lower than that of the S3G7H5 bioscaffold	The compression elastic modulus, which is equivalent to Young’s modulus in the context of compressive testing, was determined to be 8.27 MPa for the bioscaffolds. The compression elastic modulus (Young’s modulus) values were: S3: 4.13 MPa S3G7H5: 8.27 MPa	NA	NA	Tian et al., 2021 [65]
Bone and Periodontal	NA	NA	The study showed that increasing the concentration of dECM in the GelMA bioink led to an increase in the storage modulus. This means that higher dECM concentrations enhance the stiffness of the bioink, making it more suitable for applications requiring robust mechanical properties	NA	Yang et al., 2023 [70]
Periodontal	NA	NA	Stiffness of the 3D-printed scaffolds is indirectly related to the concentration of GelMA and the crosslinking process The photocrosslinked 3D-printed scaffolds using 12.5% GelMA possessed sufficient physical integrity, which suggests a certain level of stiffness that allows handling with forceps and a surgical spatula without deformation	NA	Raveendran et al., 2019 [79]
Periodontal	1% CNT/CS/AL group exhibited the highest compressive strength, suggesting that higher CNT concentrations enhance the mechanical properties of the scaffold, making it more suitable for supporting periodontal tissue regeneration	NA	NA	NA	Suo et al., 2022 [69]
Periodontal	NA	NA	NA	NA	Miao et al., 2023 [78]
Periodontal, Dentine, and Pulp	DDMp bioink: 25 kPa; PCL only: 25,000 kPa; PCL-DDMp bioink: 30,000 kPa. This highlights the importance of PCL structure in improving mechanical strength	NA	DDMp concentration has a role in enhancing the stiffness of the bioink, which is crucial for its application in dental tissue engineering	NA	Han et al., 2021 [74]
Enamel	NA	Alg4–CMC2%: 57.08 ± 6.162 kPa (highest) Alg3–CMC3%: 26.02 ± 3.663 kPa Alg2–CMC4%: 6.320 ± 1.339 kPa (lowest)	The Alg4–CMC2% scaffold exhibited the highest elastic modulus of 57.08 ± 6.162 kPa, indicating that it is the stiffest among the tested groups	NA	Mohabatpour et al., 2022 [83]
Dentine	NA	Stress-strain: CS0: 146.1 ± 6.8 kPa CS5: 193.3 ± 5.7 kPa CS10: 332.6 ± 10.4 kPa Young’s modulus: CS0: 184.9 ± 6.1 kPa CS5: 234.4 ± 8.2 kPa CS10: 413.3 ± 12.5 kPa Specifically, the Young’s modulus increased with higher concentrations of CS, with C0 (no CS) having a modulus of 184.9 ± 6.1 kPa, CS5 (5% CS) having 234.4 ± 8.2 kPa, and CS10 (10% CS) reaching 413.3 ± 12.5 kPa	Stiffness (Young’s modulus): The stiffness of the CS/GelMa scaffolds was evaluated using stress–strain curves and Young’s modulus. The presence of calcium silicate (CS) significantly increased the stiffness of the scaffolds	The CS10 scaffold, in particular, demonstrated a steeper stress–strain curve, indicating it could withstand higher strain compared to other samples. This suggests improved tensile resilience with increased CS content	Lin et al., 2021 [82]
Pulp	NA	Young’s modulus: GA: 35 ± 9.41 kPa (significantly lower) GAHA: 900 kPA GAHAP: 1100 kPA	NA	NA	Anitua et al., 2022 [84]
Soft tissues in the dentoalveolar region	NA	NA	NA	NA	Amoli et al., 2022 [81]
Dental tissues	The load failure generally occurs for strains between 0.5 and 0.7, with maximum stresses at rupture varying from 15 to 100 Kpa	NOP: 9.0 ± 1.4 kPa RDP 13.0 ± 1.5 kPa ICP: 9.1 ± 1.5 kPa or a strain of up to 0.15	Stiffness of the engineered GelMA hydrogels (7.4–14.2 kPa) was significantly higher than reported previously for UV crosslinked GelMA hydrogels of a comparable concentration	NA	Messaoud et al., 2023 [80]
Dental tissues	The compressive breaking strength of the hydrogels increased with CNF content, reaching a maximum of 249.37 KPa at 10% CNF, which is significantly higher than the pure GEL. Breaking strengths were: GEL-5: 43.32 kPA 3–CNF/GEL-5: 89.0 kPa 5–CNF/GEL-5: 154.68 kPa 10–CNF/GEL-5: 249.37 kPa (maximum) 15–CNF/GEL-5: 172.47 kpa 20–CNF/GEL-5: 102.71 kPa Optimum CNF filling content was determined to be 10%, compressive strength of 10% CNF/GEL-5: 250 kPa	NA	NA	The maximum deformation of 10–CNF/GEL-5 was greater than that of GEL-5, indicating improved tensile resilience	Jiang et al., 2018 [68]

58S BG, 58S bioactive glass; Alg, alginate; AL, sodium alginate; AMP, amorphous magnesium phosphate; CMC, carboxymethyl chitosan; CNF, cellulose nanofibre; CNTs, carbon nanotubes; CS, calcium silicate; CS0, bioink with 0% calcium silicate powder; CS5, bioink 5% calcium silicate; CS10, bioink with 10% calcium silicate powder; DDMps, demineralised dentine matrix particles; dECM, decellularised extracellular matrix; ECM, extracellular matrix; G80T20, 80% gelatine and 20% beta-tricalcium phosphate; G60T40, 60% gelatine and 40% beta-tricalcium phosphate; G40T60, 40% gelatine and 60% beta-tricalcium phosphate; GA, gelatine and alginate; GAHA, gelatine and alginate enriched in hydroxyapatite; GAHAP, gelatine, alginate, hydroxyapatite, and plasma rich in growth factor; GA, gelatine and alginate; GAHA, gelatine and alginate enriched in hydroxyapatite; GAHAP, gelatine, alginate, hydroxyapatite, and plasma rich in growth factor; GEL, gelatine; Gel, gelatine; GelMA, gelatine methacrylate; HAMA, hyaluronic acid methacryloyl; ICP, interconnected porous; NA, not available; na-HA, nano-hydroxyapatite; NOP, non-porous; PCL, polycaprolactone; PEGDA, polyethylene glycol dimethacrylate; RDP, regular-disconnected porous; S3; bioscaffold composed solely of 3% sodium alginate; S3G7H5, bioscaffold made from 3% sodium alginate, 7% gelatine, and 5% nano-hydroxyapatite; SA, sodium alginate; and UV, ultraviolet.

## Data Availability

Not applicable.

## Abbreviations

The following abbreviations are used in this manuscript:

24 h	Twenty-four hours
3D	Three dimensional
β-TCP	Beta-tricalcium phosphate
AL	Sodium alginate
Alg	Alginate
Alg-Dent	Alginate–dentine
AMP	Amorphous magnesium phosphate
ATPS	Aqueous two-phase system
BG	Bioactive glass
BGM	Bioactive glass microsphere
BMP	Bone morphogenic protein
BMP2	Bone morphogenic protein 2
Ca+	Calcium ions
CaCl_2_	Calcium chloride
CMC	Carboxymethyl chitosan
CNFs	Cellulose nanofibrils
CNF	Cellulose nanofibre
CNTs	Carbon nanotubes
CNT/CS/AL	Carbon nanotube/chitosan/sodium alginate
CS	Calcium silicate
CS	Chitosan
CS	Pure collagen/hDPSCs
CS0	Bioink with 0% calcium silicate powder
CS5	Bioink with 5% calcium silicate
CS10	Bioink with 10% calcium silicate powder
CTS-20	Collagen/20% weight percentage beta-tricalcium phosphate weight with human dental pulp stem cells
CTS-40	Collagen/40% weight percentage beta-tricalcium phosphate weight with human dental pulp stem cells
DDM	Demineralised dentine matrix
DDMps	Demineralised dentine matrix particles
DLP	Digital light processing
dECM DPSCs	Decellularised extracellular matrix Dental pulp stem cells
ECM	Extracellular matrix
EPLGMA	Glycidyl methacrylate (GMA)-modified epsilon-poly-L-lysine
ESR	Equilibrium swelling ratio
G	Gauge
G′	Elastic modulus
G″	Viscous modulus
G80T20	80% gelatine and 20% beta-tricalcium phosphate
G60T40	60% gelatine and 40% beta-tricalcium phosphate
G40T60	40% gelatine and 60% beta-tricalcium phosphate
GA	Gelatine and alginate
GAHA	Gelatine and alginate enriched in hydroxyapatite
GAHAP	Gelatine, alginate, hydroxyapatite, and plasma rich in growth factor
Gel	Gelatine
GEL	Gelatine
GelMA	Gelatine methacrylate
GTA	Glutaraldehyde
HAMA	Hyaluronic acid methacryloyl
ICP	Interconnected porous
LAP	Lithium phenyl-2,4,6-trimethylbenzoylphosphinate
MA-C	Maleic anhydride-grafted chitosan
mBMSCs	Mouse bone marrow mesenchymal stem cells
MDSC-MVs	Myeloid-derived suppressor cell membrane vesicles
NA	Not available
na-HA	Nano-hydroxyapatite
NOP	Non-porous
ODM	Osteogenic differentiation medium
PBS	Phosphate-buffered saline
PCL	Polycaprolactone
PEGDA	Polyethylene glycol dimethacrylate
PDGF	Platelet-derived growth factor
PDL	Periodontal ligament
PDLSCs	Periodontal ligament stem cells
PEI	Polyethyleneimine
PEG	Polyethylene glycol
PEGDA	Polyethylene glycol diacrylate
P.g	*Porphyromonas gingivalis*
PLGA	Polylactic-co-glycolic acid
RDP	Regular-disconnected porous
S3	Bioscaffold composed solely of 3% sodium alginate
S3G7H5	Bioscaffold made from 3% sodium alginate, 7% gelatine, and 5% nano-hydroxyapatite
SA	Sodium alginate
SCAPs	Apical papilla stem cells
SEM	Scanning electron microscope
SHEDs	Exfoliated deciduous teeth stem cells
SLA	Stereolithography
SR	Swelling ratio
Tsol-gel	Temperate at which hydrogel transitions from a sol (liquid-like) state to a gel (solid-like) state
UV	Ultraviolet
*w*/*v*	Weight/volume
wt%	Weight percentage
